# Semi-Implantable Bioelectronics

**DOI:** 10.1007/s40820-022-00818-4

**Published:** 2022-05-28

**Authors:** Jiaru Fang, Shuang Huang, Fanmao Liu, Gen He, Xiangling Li, Xinshuo Huang, Hui-jiuan Chen, Xi Xie

**Affiliations:** State Key Laboratory of Optoelectronic Materials and Technologies, Guangdong Province Key Laboratory of Display Material and Technology, School of Electronics and Information Technology, Sun Yat-Sen University, Guangzhou, 510006 People’s Republic of China

**Keywords:** Semi-implantable bioelectronics, Cell applications, In vivo applications

## Abstract

Developing techniques to effectively and real-time monitor and regulate the interior environment of biological objects is significantly important for many biomedical engineering and scientific applications, including drug delivery, electrophysiological recording and regulation of intracellular activities. Semi-implantable bioelectronics is currently a hot spot in biomedical engineering research area, because it not only meets the increasing technical demands for precise detection or regulation of biological activities, but also provides a desirable platform for externally incorporating complex functionalities and electronic integration. Although there is less definition and summary to distinguish it from the well-reviewed non-invasive bioelectronics and fully implantable bioelectronics, semi-implantable bioelectronics have emerged as highly unique technology to boost the development of biochips and smart wearable device. Here, we reviewed the recent progress in this field and raised the concept of “Semi-implantable bioelectronics”, summarizing the principle and strategies of semi-implantable device for cell applications and in vivo applications, discussing the typical methodologies to access to intracellular environment or in vivo environment, biosafety aspects and typical applications. This review is meaningful for understanding in-depth the design principles, materials fabrication techniques, device integration processes, cell/tissue penetration methodologies, biosafety aspects, and applications strategies that are essential to the development of future minimally invasive bioelectronics.

## Highlights

- Concept of “Semi-implantable Bioelectronics” is raised to cover the major advances and emphasize new insights into building external device.
- The principle and strategies of semi-implantable device for cell applications are summarized to discuss the typical methodologies to access to intracellular environment by cell penetration and various efficient applications.
- The principle and strategies of semi-implantable device for in vivo applications are highlighted to discuss the various types of transdermal devices, brain electrodes and microneedle devices for the applications.

## Graphical abstract

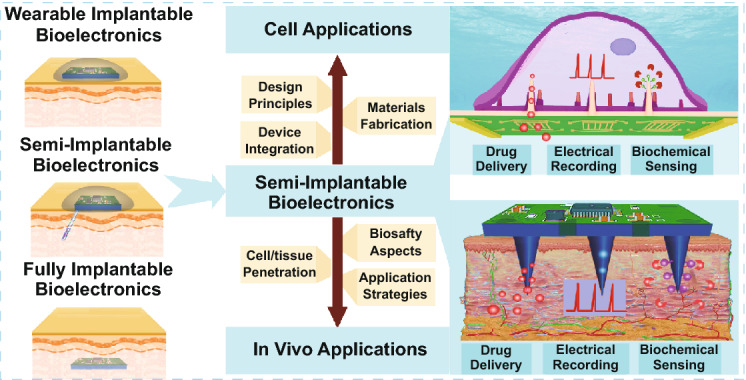

## Introduction

In modern society, health monitoring and disease treatments are extensively concerned by human. To improve life quality, personalized healthcare emerges to optimize the diagnosis and treatment according treatment according to individual individual person’s unique state, which would improve the therapeutic efficacy and reduce the adverse effects. With the personalized preventive care or personalized drug therapy strategies, the efficacy and cost of healthcare can be well regulated to benefit the individual patient. To achieve this goal, real-time, in situ detection and regulation of bioinformation is of great importance to understand the life science and develop the new generation technologies of diagnosis and treatment. Precise acquisition of bioinformation, such as detection of the specific physiological biomarkers or indicators, facilitates to guide the optimization of diagnosis and treatment strategies for the specific patients. Chip-based sensors have achieved promising progress in collecting information of body and extracellular fluid. These sensors are designed for the detection of DNA, RNA, peptides, proteins, ions, metabolites, and other bioactive molecules. For example, the gene chip and protein chip-based sensors are powerful to perform the genomes or proteomics analysis for body or extracellular samples in a simultaneous, efficient and accurate manner, and these chip sensors have been widely applied in disease diagnosis, drug screening, personalized medicine, environmental monitoring, bioinformatics, and other related fields. However, these biochips usually detect the samples by extraction of biofluid such as blood sampling, or from tissue by biopsy, which hampers the real-time and in situ information detection.

The rapid developments of wearable and implantable electronics have also paved a promising way for the personalized and precise medicine. Nowadays, flexible and stretchable characteristics of electronics are emerging development trends, where non-invasive and implantable electronics are often self-adaptive and conformal to couple on the curved and deformable tissue surface, which is of great superiority compared to the conventional rigid planar electronics. With the advanced micro/nanofabrication technologies and versatile functional materials, the high-throughput, multiplexed, high-sensitivity, and miniaturized biosensors are developed and integrated into non-invasive wearable or implantable devices to continuously monitor the physiological and biochemical signals. Meanwhile, the portable and miniaturized wearable or implantable bioelectronics provides promising strategies for personalized healthcare and precise therapy, which are both based on continuous monitoring and diagnosis. Remarkably, the real-time closed-loop regulation is the advanced characteristic and function of wearable non-invasive and implantable electronics.

Wearable device is often noninvasive and biocompatible to collect bioinformation from the body surface. The noninvasive electrophysiology recording systems for ECG, electrocorticogram (EEG), or EMG are successful cases of applications of wearable device, and these bioelectrical signals can greatly facilitate the acquisition of information to directly reflect the health and function status of heart, brain, or muscle. In addition to medical devices, noninvasive wearable recording systems (e.g., smart watch, smart band) are attractive hotspots in recent decade for the monitoring of biophysical and biochemical signals (ECG, pulse, blood pressure, bioactive molecules, and other metabolites) from living body. Meanwhile, commercial implantable devices, such as fully implantable glucose sensors, generally including sensor probe, electronics modules, data transmission module, and battery in an integrated device, can provide real-time monitor of blood glucose level and support the extensive application for months. Heart pacemaker is another successful case of implantable device, which consists of pulse generator or electrode wires. By rhythmically stimulating the local cardiomyocytes, the heart pacemaker facilitates electrical signals to spread to the entire heart, which could regulate the functions of contraction and blood pumping, while the electrode wires also transmit the ECG of heart cavity to pack-maker device for feedback control.

In spite of the early success of wearable device and implantable devices for health regulation by real-time diagnosis and close-loop treatment, the functional scope of them is still suggested to inherit limitations. The wearable devices conventionally attached on the outer surface of skin can only measure the physiological signals on the body surface, such as ECG, pulse, or blood pressure. The sweat-based biomarker detection relies on sufficient sweat samples, and the result accuracy is insufficient to reflect the actual states of analytes in blood or interstitial fluids, which is rarely able to actually achieve the disease-related information in the body. On the other hand, the implantable devices can record the information deeply inside the body, while the power supply, volume, and material safety of implantable devices are demanded to be developed for the successful and further application. In addition to the devices for human body monitoring, a large number of devices are designed to record the electrophysiological and biochemical signals of cells. Though the micro- or nanodevice can record the weak extracellular electrical signals or trace biomarkers of cells, the extracellular information is insufficient and inaccurate to reflect the physiological status of cells. For example, the electrophysiological devices can only record the extracellular potentials rather than intracellular ones, and signals distortion cannot be avoided due to passing through the cellular membrane. The electrochemical devices can only detect the metabolites in the extracellular microenvironment, and rarely able to accurately reflect the intracellular metabolism. Taking these limitations of noninvasive and implantable device into account, the forms of device between wearable and implantable device may be an effective complement for these potential shortcomings.

Here we raise the concept of “Semi-implantable bioelectronics,” referring to functional or electronic devices that could access to the interior environment of biological objects such as cells, tissues, or animal/human bodies, while the connected bulk devices remained on the surface of the biological objects. Compared with other bioelectronics, such as wearable bioelectronics or fully implantable bioelectronics, semi-implantable bioelectronics establish a platform to precisely detect or regulate biological activities insides biological objects and perform externally incorporated functionalities by electronic integration. In the past decade, we witnessed the significant experimental and theoretical advances on various types of semi-implantable bioelectronics, yet they have not been clearly defined and summarized to distinguish them from the well-reviewed wearable bioelectronics and full-implantable bioelectronics.

With the rapid technologies’ advances of bioelectronics, the semi-implantable bioelectronics based on nanoneedles for cell penetration have played important role in the recording and regulation of intracellular activity. Cell is separated from the outside environment by phospholipid bilayer, limiting the access of external to the rich information inside the cell. To investigate the intracellular activities, it is necessary to reach the inside the cell through the phospholipid bilayer. Meanwhile, cell lipid bilayer is also a barrier for external tools to explore the intracellular biological and physiological events [1]. The conventional extracellular recording technology collects low-quality biochemical and electrophysiological signals due to the barrier of cell membrane, and extracellular drug delivery suffers from low efficiency and large cell damage [2–7]. Consequently, it is urgently demanded to seek the safe and efficient intracellular operation or recording approaches. Due to the unique properties to pierce the cell membrane based on nanoneedle structures [8–14], semi-implantable bioelectronics present effective cell penetration approaches for the biochemical and biophysical access, so that the high-efficiency molecule/drug delivery and high-quality intracellular electrophysiological/biochemical sensing could be readily achieved [15–17].

The cell penetration efficacy of semi-implantable devices is realized by spontaneous penetration [18–20] or artificially assisted penetration, e.g., based on chemical coating [21–23], electroporation [24–26], mechanical force [27, 28], or optoporation [29, 30]. Once the semi-implantable device penetrates cell membrane, a large amount of intracellular sensing and regulating operations (e.g., electrical recording [31, 32], biochemical sensing [33–35], and drug delivery [36–38]) can be readily performed. For intracellular operation, the semi-implantable devices have emerged as the powerful tools that have attracted a broad research interests.

In addition to the in cellular application, with the rapid technologies’ advances of biocompatible materials and flexible electronics, semi-implantable electronic devices present promising prospect in real-time and continuous monitoring of multiple physiological parameters in vivo, involving detection of basic electrophysiology and biochemical markers. Due to the semi-implantable feature, issues associated with skin barrier, signal integrity and power supply can be effectively addressed, while these limitations and restriction have been widely encountered by conventional noninvasive device or fully implantable devices. The investigation of semi-implantable bioelectronic system can not only provide new opportunity to monitor the biomarkers of interstitial fluid (ISF) and record electrophysiology of subcutaneous tissues by the semi-implantable sensors, but also deliver drugs and apply optical/electrical stimuli to the target tissues by the semi-implantable stimulating probe/tubing. For example, the commercial continuous glucose monitoring devices are usually semi-implanted into the body, remaining the measurement, data transmission, and power modules outside the body [39–41]. Compared with wearable noninvasive devices, the accuracy of glucose measurement based on this semi-implantable strategy is greatly improved. Compared with fully implantable devices, the power durability and data transmission integrity are effectively ensured by integration with external module on body surface, so that frequent surgical operation could be avoided. Consequently, the semi-implantable devices combine the advantages of wearable noninvasive and implantable devices, which are successful to achieve a wide variety of applications in vivo and meet the clinical applications. Based on the accurate measurement results of semi-implantable strategy, close-loop function (refers to the processing with a feedback component, which makes the detection more accurate through a feedback system) can be reliably perform for controlled drug delivery, which could prompt the semi-implantable strategy into highly intelligent device. On the other hand, the electrophysiological signals in vivo using semi-implantable devices mainly include electrocorticogram (EEG) and intracortical signals without filtering by skull, so they could record more accurate signals than the noninvasive EEG [42–44]. To record the high-throughput and high-quality signals, a large number of semi-implantable brain electrodes serve as utility tools for the electrical signal recording under the skull. In addition to the signal recording function, the regulating functions such as drug delivery and optical/electrical stimulation are also developed and integrated in the semi-implantable devices [45–47]. For the in vivo application, the semi-implantable bioelectronics will be an optimal option taking the accuracy, practicability, and safety into account.

In this comprehensive review article, we will raise the concept of “Semi-implantable Bioelectronics,” cover the major progresses with the most general applicability and emphasize new insights into the development of building external device that could access to the interior environment of biological objects to precisely detect or regulate biological activities. To do so, we will first summarize the principle and strategies of semi-implantable device for cell applications, discussing the typical methodologies to access to intracellular environment by cell penetration and their biosafety aspects, and various efficient applications including drug delivery, biochemical sensing and electrical recording insides cells. Then, we will highlight the principle and strategies of semi-implantable device for in vivo applications, discussing the various types of transdermal devices, brain electrodes and microneedle devices for the applications including electrical recording, biochemical sensing, drug delivery, and stimulation in vivo. This summary of design principles, materials fabrication techniques, device integration processes, cell/tissue penetration methodologies, biosafety aspects, and applications strategies outlines the potential of Semi-implantable Bioelectronics as a practical biomedical engineering solution.

## Semi-Implantable Device for Cell Applications

### Principle and Strategies

Cell is enclosed by a phospholipid bilayer called plasma membrane, which effectively insulates the internal from external microenvironment and maintains the homeostasis [1]. It is important to regulate the cell behavior for emerging biomedical research (e.g., gene editing), while these types of tools lead to attractive research hotspots to explore the interior of live cells through the plasma membrane [48–50]. In most cases, the cell membrane with thickness of ~ 6 nm is an insurmountable barrier due to the unique lipid bilayer structure, preventing most aqueous compounds and probes from reaching the intracellular region. Therefore, cell membrane penetration methodologies for intracellular operations are a focused mission in the scientific research and therapeutic applications (e.g., electrophysiological recording, biochemical detection, and molecular delivery). In the recent decades, a large amount of nanodevices emerge by advanced micro/nanofabrication technology to facilitate accessing to the in-cell area in a semi-implanted way [51–54]. Particularly, one-dimensional nanostructures (e.g., nanowires, nanowires, nanotubes, nanopillars) enable penetration of the cell membrane by local high pressure of their sharp nanotips [55–58]. The functions of these nanoscale platforms for cell penetration can be based on nanoarray or individual functional nanostructure. Atomic force microscopy (AFM) or micromanipulators are common tool to assist single nanoprobe devices (e.g., nanowire) to record and regulate the intracellular activities [59, 60]. It is meaningful to perform the high spatiotemporal resolution sensing and operating in single cells for exploring the cellular micro/nano-environment, detecting intracellular biochemical indicators, and revealing the intercellular differences at a subcellular/nanoscale resolution.

However, the wide applications of single nanowire platforms are hindered due to low throughput, which is difficult to synchronously perform the operation for numerous individual cells [61, 62]. To solve this limitation, vertical aligned nanowire arrays provide a unique platform for the large-scale and high-throughput of cells with even single-cell manipulation [63, 64]. Spontaneously, the cell-nanostructure-interface can form locally higher tension on cell membrane based on the cellular force at the sharp nanotips [65, 66]. In the practical applications, vertical nanowire devices can even penetrate the cell membrane based on their 3D sharp feature. However, this spontaneous penetration usually presents low-efficiency, and consequently, chemical coating [21–23], electroporation [24–26], optoporation [29, 30], or mechanical force [27, 28] are often introduced to assist the cell penetration. Nanowires that penetrate cell membrane can form a semi-implanted profile into the cell, and various intracellular applications (e.g., electrophysiological recording [31, 32], biochemical sensing [33–35], and molecule delivery [36–38]) can be versatilely carried out. For the electrophysiological and biochemical recordings, semi-implantable nanoprobes can directly contact with cytosolic contents, which allows high-sensitivity detection of the intracellular electrophysiological and biochemical signals. For the molecule delivery, the various nanowires-based semi-implantable device can efficiently achieve synchronous delivery into a large amount of cell types [18, 67], which effectively avoids the endocytotic degradation of conventional molecule delivery methodologies. To improve the practicability of these semi-implantable nanodevice, it is of great significance to deeply explore the mechanism of cell–nanowires interactions [68–70].

### Materials and Devices

#### Single Nanowire Platform

**Fig. 1:**
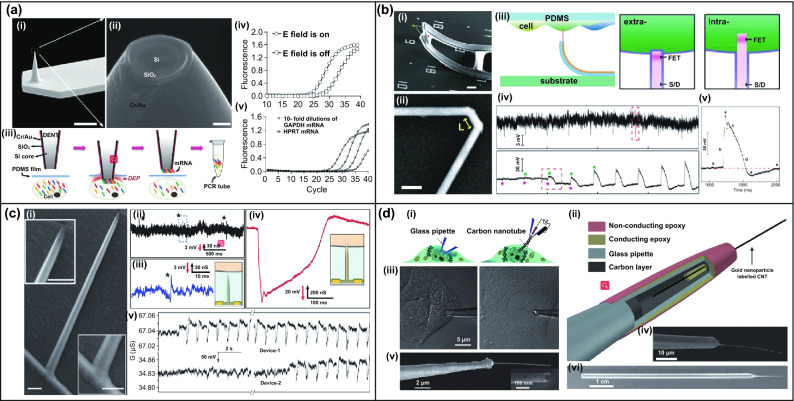
**a** (i, ii) SEM images of the coaxial AFM probe. (iii) The operation of single-cell mRNA extracted using DENT. (iv) Figure indicates field on and field off for β-actin mRNA. (v) Detection of GAPDH and HPRT [71–73]. **b** (i, ii) SEM images of an as-made device and a doubly kinked nanowire with a cis configuration. (iii) Diagram of recording from the monolayer of cardiomyocyte. (iv) Extracellular and intracellular electrical recording from beating cardiomyocytes. (v) Steady-state intracellular recording [74]. **c** (i) SEM image of a nanotube on a silicon nanowire. (ii–iii) Representative trace reflecting the extracellular recording. (iv) Magnified view of the peak in the intracellular recording. (v) Simultaneously recording extracellular and intracellular electrical signals from the two devices [75]. **d** (i, ii) Comparison of cellular endoscopes with glass pipettes. (iii) HeLa cell (left) was interrogated by a glass pipette, and a primary rat hepatocyte nucleus was interrogated by a nanotube endoscope. (iv, v) SEM image of endoscopes fitted with 100 and 50 nm carbon nanotube tips, respectively. (vi) Optical image of a glass pipette with a carbon-nanotube tip [59]. Reproduced with permission from Refs. [59, 71–75], copyright (2010) Nature Publishing Group (2017) Applied Physics Letters Publishing Group (2017) The Royal Society of Chemistry Publishing Group (2009) Applied Physics Letters Publishing Group (2010) American Association for the Advancement of Science Publishing Group and (2011) Nature Publishing Group

For the intracellular access, single nanowire-equipped cantilevers are employed for the probing intracellular environment with high spatial resolution at single cell resolution, and nanoscale probes also greatly reduce the interference and damage of cell during membrane penetration. These 1D nanostructure-based device is semi-implantable into cells, with a tiny probe in diameter of 1–1000 nm assembled on a supporting pipette or cantilever. Due to the nanoscale size and semi-implantable property, the nanoprobe allows the precise and long-term positioning into cell, where the nanoprobes can be consisting of various materials, such as carbon nanotubes, silicon nanowires, and gold nanopillars. Due to the 3D operation demand, AFM is a common assembly platform for nanoprobe to guide the probe to the cell position and to complete cell penetration. Nawarathna et al. developed a heavy-doped silicon nanoprobe, named as dielectrophoretic nanotweezer (DENT), to extract RNA and DNA from targeted cells [71–73]. The conductive silicon nanoprobe surface was insulated by a SiO_2_ layer, and metal layer (Cr/Au) was then deposited on the passivation layer. To extract nucleic acid molecule, a 120 kHz AC, 5 V_PP_ AV sine voltage are applied between the core and surface conductive layers for ~ 1 min, where a strong dielectrophoretic force was generated to adsorb nucleic acid molecules at the DENT nanotip (Fig. 1a). The nucleic acids can be extracted from the cytosol and transferred into the polymerase chain reaction (PCR) for amplification, and thus, the DENT-integrated AFM probe allows nanoscale resolution positioning of targeted cells. Besides, the nanoprobe could maintain the good viability of the living cell with minimally destruction. The detection can be extended for specific collection of targeted mRNAs, where specific-primers could be functionalized on the DENT for enrichment of mRNA.

For the investigation of intracellular electrophysiological signals, patch clamp is a powerful tool which enables recording of single ion channel behavior. However, the low throughput and complicated operation of patch clamp are main shortcoming during large-scale synchronized study of individual cell. To overcome these drawbacks, Lieber et al. developed 3D nanoFETs by advanced silicon nanowire (SiNW) fabrication to record intracellular electrical signals of excitable cells. The essential component of the device contained kinked NWs to form a 3D nanoFET architecture [74], where the bend-up kinked structure enabled cell membrane penetration with the assistants of phospholipid modification to facilitate membrane rupture (Fig. 1b). The 3D kinked nanoFET initially recorded the extracellular action potentials, and the extracellular signals gradually changed into intracellular action potentials with amplitude of ∼80 mV, which have similar amplitudes with the gold-standard signals (70–100 mV). Based on the similar principle, branched SiO_2_ nanotube-based 3D FET (Fig. 1c) was developed in 2012 by the same group [75], which could also penetrate into cardiomyocytes by a phospholipid bilayer coating on the nanotube surface. After rupture of cell membrane, the cytosol could infuse and contact to the SiNW FET through the nanotube, so that intracellular action potentials can be subsequently recorded by the potential changes of the electrolyte gate.

Besides detection of cellular electrical signals, single nanoprobe-based semi-implantable device could also enable detection of biochemical molecules within cells. While glass micropipettes are common tools for the suction of cell membrane, the large rupture size on membrane of micropipettes-based devices may damage the cells during penetration or detection. Singhal et al. invented nanoendoscope for intracellular biochemical detection and regulation at the single organelle resolution (Fig. 1d) [59], employing ultra-sharp multiwalled carbon nanotube possessing diameter of ~ 100 nm and length of 50–60 mm as the essential cell penetration substrate. Owing to the high spatial resolution of the small nanotube structure, this nanoendoscope can either access to the intracellular environment of a single cell for biosensing, or even analyze the interior of sub-cell organelles. Moreover, the hollow nanotube on the nanoendoscope could serve as a nanochannel for intracellular drug delivery or extraction of cytosolic molecules. Since the single nanoprobe-based semi-implantable device match the size of single cell, it is a promising tool to perform intracellular probing and stimulation, although the operations need multiple steps of alignment and micro-manipulation.

#### Nanowire Array Platform

Nanowire array can serve as powerful semi-implantable platform to treat multiple cells simultaneously. Due to the low throughput of single nanowire platform, nanowires aligned in array could improve the throughput of interfacing with multiple cells. The cells could be cultured on top of nanowire array in vitro, where the nanowires could penetrate cell membrane with assistance of poration techniques, so that the cytosolic content could be accessed. Nanowires can be prepared by a large number of material types, where silicon is a common nanowire material for intracellular exploration with good biocompatibility and stability in microenvironment of biological cells [76–78]. Other materials, (e.g., GaP [79], InAs [80], SiO_2_ [81, 82], Al_2_O_3_ [83], ZnO [84], SnO_2_ [85, 86], carbon [87], diamond [88], and Pt [89, 90]) also present good biocompatibility to cells, and the advanced nanowire fabrication technology can now tune the structures, sizes, compositions, and physical/chemical properties of nanowires in well-controlled manner. These characteristics enable the versatile functions of nanowire array for electronic, photonic, and magnetic applications besides biological cells [91–95].

**Fig. 2:**
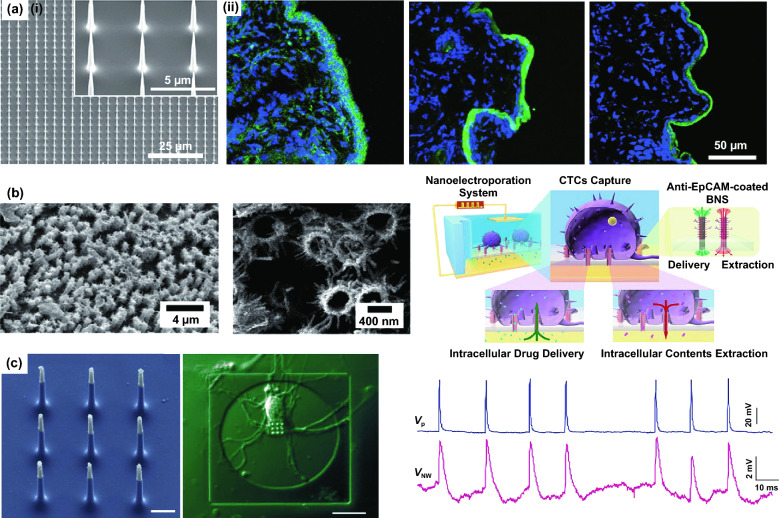
**a** (i) SEM image of the silicon nanowire array. (ii) Fluorescent images showing tissue sections of nanowire arrays + TENG, Flat + TENG and nanowire arrays after treatment with 10 kD dextran- FITC delivery [98]. **b** SEM images of the BNS array, and schematic diagram of cell delivery and extraction in situ after capture of cancer cells by an electroporation system constructed with BNS array [84]. **c** SEM images of the VNEA and DIC micrographs of rat cortical neurons after incubation on them. Action potentials were stimulated with a patch pipette (blue), and also recorded by the VNEA pad in Faradaic mode (magenta) [101]. Reproduced with permission from Refs. [84, 98, 101], copyright (2019) American Chemical Society Publishing Group (2019) Wiley-VCH Publishing Group and (2012) Nature Publishing Group

For applications of intracellular molecule delivery, solid nanowire array-based semi-implantable devices are constructed to penetrate cell membrane and to mediate the transportation of biomolecules into cells in vitro by drug elution from nanowire surface [96, 97]. In recent work, a self-powered nano-electroporation system was established on the basis of a triboelectric nanogenerator (TENG), and this nanowire array-based electroporation system can achieve intracellular delivery in vivo (Fig. 2a) [98]. TENG-based nano-electroporation system harvested energy by body movements and subsequently generated electric field on the nanowires, which would porate cell membrane to allow high-efficiency of drug delivery into cells in vivo. In addition to solid nanowire arrays, hollow nanowire arrays with unique nanochannels, which were called “nanostraws,” could directly bridge the external reservoir to intracellular environment and facilitate the delivery into target cells [99, 100]. In our recent work, vertical nanostraw array was fabricated with branched nanospikes, which was employed to deliver biomolecules into captured circulating tumor cells (CTCs) for in situ regulation or re-program of the cells (Fig. 2b) [84]. The nanostraws could porate cell membrane with high efficiency by coupling with electroporation and thus, allow gene delivery into cells, and extract the intracellular cytosol from cells with minimally invasiveness.

For high-quality electrical signal recording, nanoelectrode arrays pave a new way for intracellular electrophysiological recording by minimally invasively penetrating cell membrane. Robinson et al. established vertical nanowire electrode array (VNEA) by advanced ‘top-down’ nanofabrication (Fig. 2c) [101], where each nanowire electrode with the diameter of ~ 150 nm consisted of a conductive Si core and an insulated SiO_2_ shell, and the nanotip was sputtered with a conductive Ti/Au layer. The VNEA formed a semi-implanting patch to probe the intracellular action potential. With optimal nano-electroporation condition, the VNEA could achieve synchronous intracellular electrical stimulating and recording of neurons. In these systems, the cell membrane penetration by nanowires is the key step for achieving semi-implantable access to the intracellular environment.

### Cell Penetration Methods

**Fig. 3:**
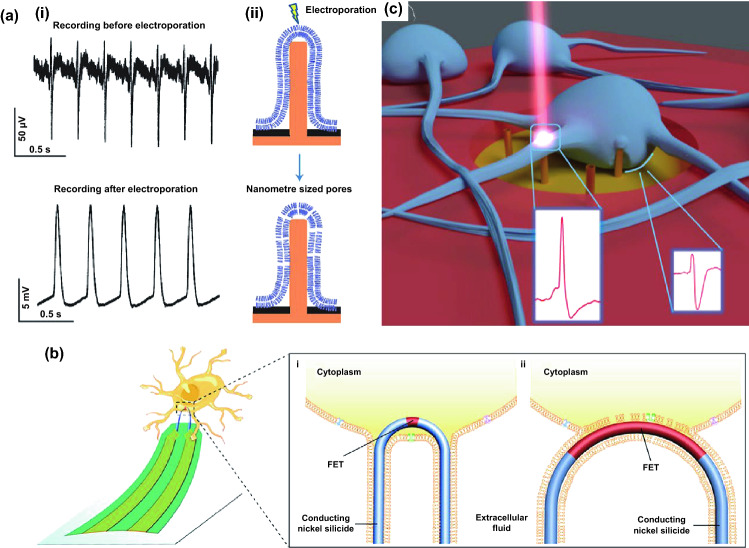
**a** (i) Before electroporation, action potentials recorded shows extracellular signatures. After electroporation, the intracellular signal amplitude increases above 100-fold. (ii)Schematic of the electroporation of the cell membrane by a nanopillar electrode [90]. **b** Diagram of intracellular recording by a U-NWFET probe. (i) An internalized short-channel U-NWFET with a high resistance seal on the cell membrane for high amplitude recording. (ii) Partially closed/internalized U-NWFETs have longer channel length/ROC leading to weaker intracellular-like action potential recordings [106]. **c** Neurons cultured on MEA with 3D nanoelectrodes. 3D nanoelectrode recorded intracellular activity, while the other electrodes recorded extracellular signals [30]. Reproduced with permission from Refs. [30, 90, 106], copyright (2017) American Chemical Society Publishing Group (2012) Nature Publishing Group and (2019) Nature Publishing Group

The cell-nanowire interfaces are attractive research hotspots to understand the interaction between nanowires and cells, and to optimize the efficiency of cell penetration, yet the mechanism of nanowire cell penetration is still not fully revealed. Spontaneous penetration theory has revealed that part of nanowires initially penetrates into cell due to the sharp physical geometry of nanowires, and the gravity or adhesion forces of cells generates locally higher tension to induce the membrane penetration [65, 66]. While many studies have proved the feasibility of spontaneous penetration and semi-implantable cellular access by nanowires [18–20], the efficiency of spontaneous penetration rate is observed to be low and limits the intracellular access. To improve penetration efficiency, other assisting strategies such as electroporation [83, 90], optoporation [29, 30], chemical coating [21], or mechanical forces [102] are developed to enhance cell penetration. Many works have demonstrated that lipid membranes can be permeated by the applications of external forces (e.g., force, electricity, and light) as well as by chemical agents to be coupled with vertical nanowires devices. Three main assisting strategies of coupling external forces for cell penetration possess their own advantages and disadvantages. Electroporation coupling can lead to the induction of nanopores in cell membranes, but may disrupt cellular activity, and the resealing of nanopores can hamper long-term recording. Optoporation coupling with vertical nanowires can prevent the defect of electrical interference, but exists the problem of low-throughput regulation. The application of external mechanical forces with nanowires on cells appears to be a direct method to increase membrane penetration, but the penetration efficiency is relatively lower than the electroporation or optoporation methods. For example, Xie et al. developed nanopillar-electroporation system by focused ion beam. Initially, no intracellular potentials can be recorded by culturing cardiomyocytes on the Pt nanopillars, demonstrating no spontaneous penetration (Fig. 3a). When low voltage electroporation was applied on the Pt nanopillars, nanopores were introduced in the plasma membrane, and high-quality intracellular potentials were recorded for several minutes. Moreover, these nanopillar-electroporation system can be performed on the same cell repeatedly in continuous days. In addition to electroporation, phospholipid bilayer coating on nanowires is another assisting penetration method to improve membrane rupture by membrane fusion with coated nanowires [23, 103]. The chemical coating penetration strategies have previously been applied on single nanotubes or kinked nanowires devices to record intracellular potentials [75, 104, 105]. In recent work, Zhao et al. designed a U-shaped nanowire FET arrays [106], where high-quality intracellular potentials of neurons were recorded by inserting the nanowire FET into cells (Fig. 3b). Furthermore, optoporation is also proposed as effective method to improve nanowire cell penetration, where focused high-power laser pulses at the cell-nanowire interface are introduced to porate cell membrane, allowing simultaneously recording of both extracellular and intracellular potentials from neurons and cardiomyocytes (Fig. 3c).

### Cell Safety

**Fig. 4:**
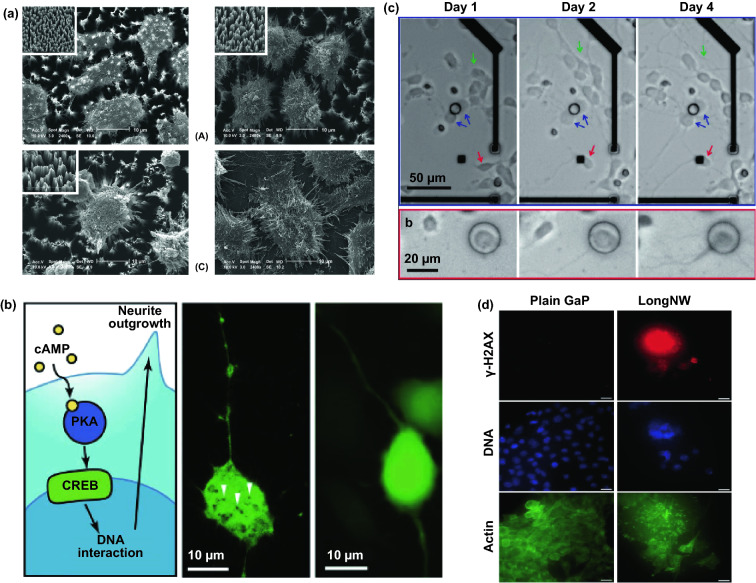
**a** Morphology and diffusion of cultured hepatic cells on Si NW arrays with different NW density. Scale bar: 10 µm [110]. **b** Maturation of neurons on InAs NW array [108]. **c** The migration of cortical neurons is followed at 1, 2 and 4 days after plating [111]. **d** γ-H2AX assay to study DNA double-strand breaks [112]. Reproduced with permission from Refs. [108, 110–112], copyright (2011) Wiley–VCH Publishing Group. (2009) American Chemical Society Publishing Group (2010) American Chemical Society Publishing Group and (2013) Wiley-VCH Publishing Group

Semi-implantable nanowires-based devices have been widely utilized as regulating tools for cells in a precisely spatial resolution and low-perturbation manner. Initially, the main indicators to assess the perturbation of nanowires-device on cells are chronic cell viability and cell membrane integrity. The safety of semi-implantable nanowire devices was evaluated by culturing different cell types with various nanowire materials in vitro. In one study, cell viability was examined by culturing mouse embryonic stem cells and HEK 293 T cells on SiNW arrays [107], where the cell viability was observed to be about ~ 78% after 3 days culturing on 90-nm-diameter and 6-µm-height NWs, where increase in NW diameter seemed to induce lower viability. Hӓllstrӧm et al. demonstrated neurons cultured on GaP nanowires possessed higher viability than those cultured on planar substrates. In addition to the cell viability, more comprehensive cellular functionality involving cell adhesion, enzyme activity, membrane protein expression, mRNA expression, the maturation pathway, etc., after cultured on short and thin NWs (e.g., ~ 100 nm diameter, < 3 µm height) was investigated (Fig. 4b) [108, 109], where the results demonstrated that the shorter nanowires minimally affect the basic cell viability and functionality.

In addition to the safety evaluation, the cell behaviors on nanowire devices have also been explored. Based on the morphology observation and biological active molecule analysis, it is found that Si nanowire arrays improve the cell attachment, while cell migration and spreading are also restrained (Fig. 4a) [110]. Similar results have also been observed in other study, where nanopillars tightly fixed the cells and restrict cell mobility (Fig. 4c) [111]. Besides, Persson et al. found that the longer nanowires (e.g., ~ 7 µm height) could negatively impact cells, by inducing high stress and high respiration rates for cells, leading to reactive oxygen species elevation and gene damage (Fig. 4d) [112].

### Applications

Efficient cell penetration by semi-implantable nanowire devices enables a large amount of biomedical research and practical applications, including electrophysiological recording, biochemical sensing, and biochemical detection. Nanowire penetration facilitates the bridging between intracellular and extracellular microenvironment, enables the transportation of exterior cargo into the cytosol, and achieves the detection of intracellular information with excellent spatiotemporal resolution.

#### Molecule Delivery

**Fig. 5:**
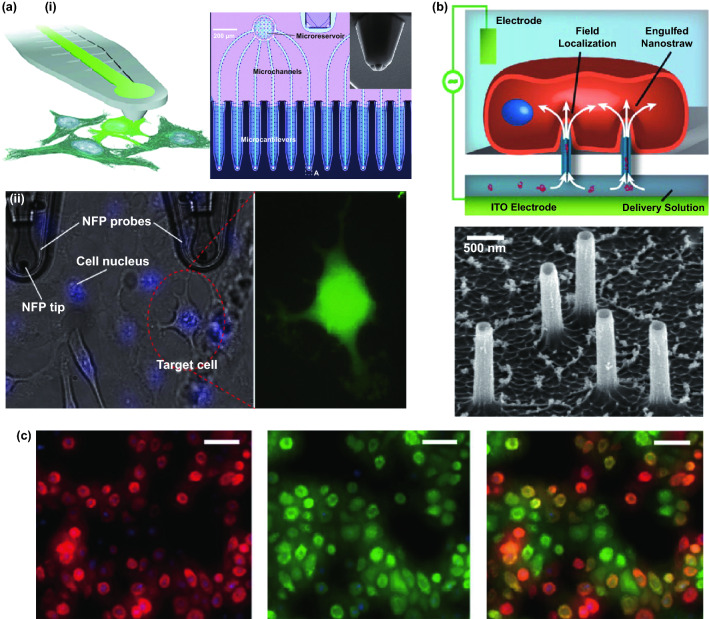
**a** (i) Optical image of NFP chip used for single-cell electroporation and SEM image of the tip of NEP. (ii) Optical images of transfection of dextran Alexa Fluor 488 into target HeLa cells with NFP tip and fluorescence images of the transfected HeLa cells [115]. **b**, **c** The tight contact between the nanostraw-plasma membrane interface allowed high-efficient transfection of pRFP [83]. Reproduced with permission from Refs. [83, 115], copyright (2013) American Chemical Society Publishing Group and (2013) American Chemical Society Publishing Group

Conventional molecule delivery relies on cell endocytosis-based pathways, yet the cargo degradation, low delivery efficiency, and cell cytotoxicity are main issues during this delivery process. In contrast to traditional delivery, semi-implantable nanowire device could mediate intracellular delivery with a broad delivery substance for various cell types. To minimalize the disturbance to cells and improve transfection efficiency, semi-implantable nanowire devices can be designed with ultra-small nanoscale feature, so that the nanostructures protruding into cells cause minimal effects to the cells [99, 113, 114]. Intracellular molecule delivery into individual targeted cells is greatly significant for diagnostics and therapeutics toward the personalized medicine in biomedical field. The bulk electroporation techniques have been conventionally employed for cell transfection in the past decade, yet the low-resolution and requirement of high voltage supply resulting in unstable molecule delivery for bulk electroporation. In contrast to conventional bulk electroporation, nano-electroporation could provide highly localized and precise electroporation on cells and thus, significantly improve poration efficiency and cell viability (Fig. 5a) [115, 116]. In theory, the nanowire-electroporation effect usually occurs at the tip of cell-nano-interface, where low voltage is sufficient to reach the critical condition for cell membrane perforation due to good coupling of cell membrane with nanostructure. For example, nanofountain probe (NFP) technology could precisely perform gentle electroporation and intracellular delivery on cells, which mediated transfection of HeLa cells with fluorescent molecules with high transfection efficiency (> 95%) and high viability (> 92%).

Although the single nanowire devices can successfully achieve high efficiency of intracellular delivery into single cell, the low-throughput feature of single nanoprobe platform due to complicated and time-consuming operations greatly imped their practical applications. To increase the delivery throughput, nanowire arrays devices are widely employed for intracellular drug delivery with high efficiency and viability [117–120], which reduces the complexity of device architecture and operations [121]. Xie et al. developed Al_2_O_3_ nanostraw-electroporation system to achieve high efficiency of transfection with high cell viability (Fig. 5b, c) [83]. The nanostraws are fabricated on a polycarbonate template followed by Cl_2_ etching and O_2_ plasma, where the diameter of nanostraws can be tunable based on different templates and fabrication conditions. In addition, the tight seal between the cell membrane and nanostraws can induce enhanced electric field on the nanostraw-cell interface, which dramatically reduced the amplitude of electroporation voltage for porating cell. It is worth noting that the delivery efficiency will be affected by cell types, such as cells lines (e.g., CHO, HEK 293), and hard-transfected cells including human-induced pluripotent stem cell-derived cardiomyocytes (hiPSC-CMs), human embryonic stem cells (HSCs), and mouse primary neuron cells (MNs). [67]. Transfection efficiency of primary cells could reach ~ 60% to 85%, which indicates the wide applicability for transfecting various cell types. Besides, diverse biomolecules (e.g., DNA, mRNA, and proteins) can be precisely delivered into cells by the nanostraw platform with controllable dose, where the intracellular is also spatially and temporally controllable. By regulating the delivery dose of mRNA or DNA, the intracellular expression of different proteins can be well controlled over time, which paves a new way to improve the practicability of delivery by multimodal gene regulation.

#### Biochemical Sensing

Biochemical markers can indicate the cell state, yet the measurement of biomarkers based on secreted biomarkers in extracellular medium cannot effectively reflect the corresponding intracellular information. Consequently, end-point cell lysis is usually carried out for the detection of intracellular biomolecules. Meanwhile, imaging analysis methods (e.g., fluorescent dyes or functional nanoparticles) have been employed for dynamic tracking of intracellular activity [122, 123], yet label-based technologies have limited spatiotemporal resolution, phototoxicity, and chemical adverse effects. To perform dynamic and biocompatible recording, semi-implantable nanowires are developed as intracellular biosensing tools, aiming at the precise measurement of intracellular biomarkers based on their unique cell penetration capacity [33]. Compared with the conventional cell lysis, the nanodevices can directly detect biomarkers in a quantitative and sensitive way without large analytical instruments [124, 125]. Moreover, the semi-implantable nanodevices enable the repeatable extraction from intracellular content and achieve the high-throughput parallel sensing.

**Fig. 6:**
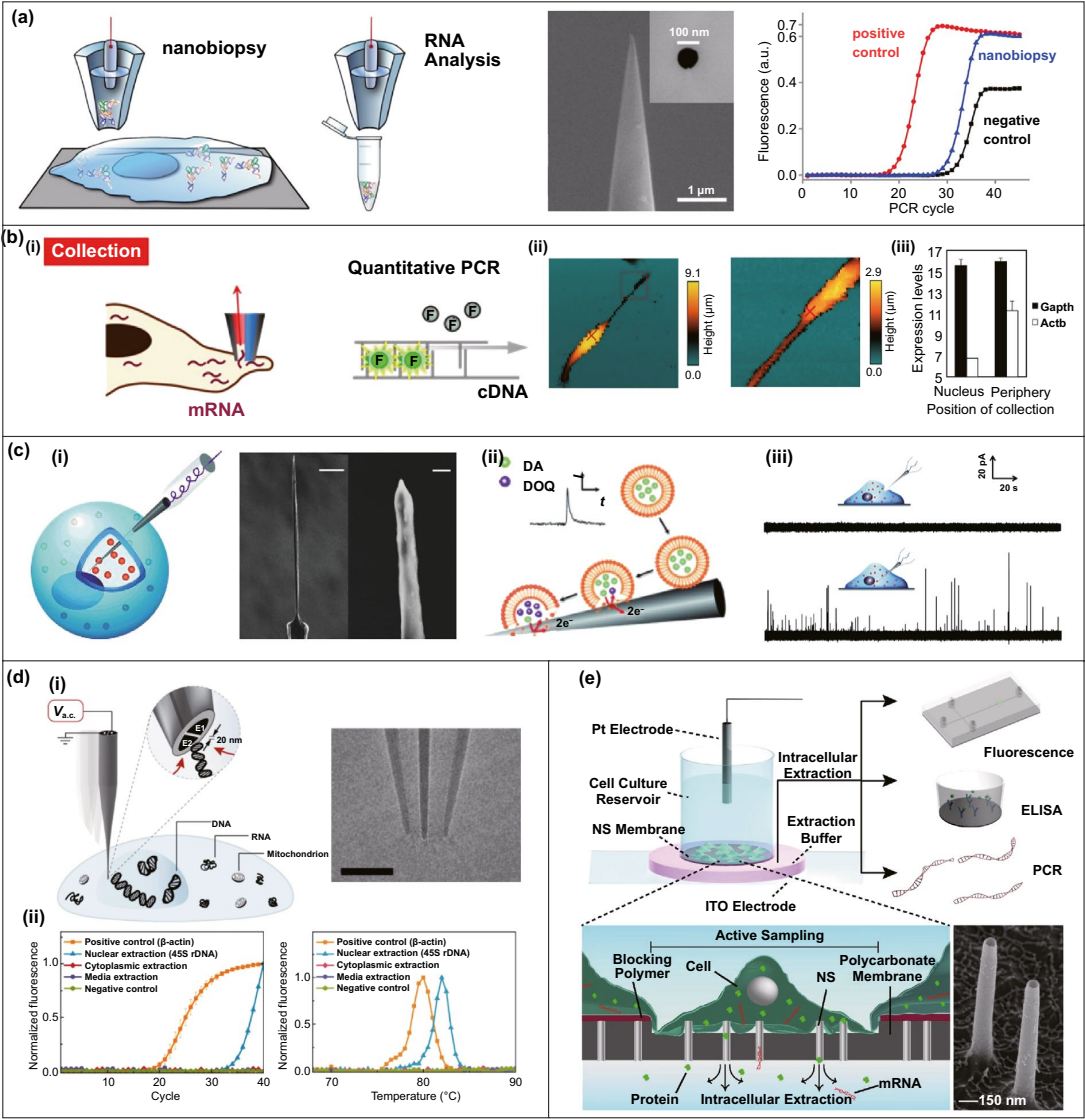
**a** A schematic of the nanobiopsy examinated by RNA analysis and an SEM image of the nanomicrotubule tip [126]. **b** (i) Schematic diagram of the electrochemical injector moving to the target location in a semi-automatic manner to collect mRNA-containing cytosol and detect it by qPCR. (ii) SICM images of the collection process. (iii) Statistical analysis of mRNA expression levels at each site [127]. **c** (i) Schematic illustration (small orange circles represent vesicles), and view of a nanotip conical carbon-fiber microelectrode. (iii) Mechanism of the adsorption and opening of vesicles on the in situ electrode. (iv) Amperometric traces for a nanotip conical carbon-fiber microelectrode pushed [128]. **d** (i) Schematic diagram of applying AC voltage to nanotweezers for targeted capture of molecules in solution or within cells, and TEM image of the DEP nanotweezer. (ii) Average qPCR amplification and typical melting curves of extracted DNA [129]. **e** The nondestructive nanopipette intracellular sampling system allowed contents to be extracted by electroporation from cells cultured on 150 nm polymer membranes and analyzed by fluorescence, ELISA or qPCR. SEM images of the NS (bottom right) [130]. Reproduced with permission from Refs. [126–130], copyright (2014) American Chemical Society Publishing Group (2016) American Chemical Society Publishing Group (2015) Wiley–VCH Publishing Group (2019) Nature Publishing Group and (2017) Proceedings of the National Academy of Sciences Publishing Group

Nanopipettes are typical semi-implantable tools which are fabricated with a submicron or nanoscale opening tip to provide a transport channel for intracellular operations. To precisely extract contents from targeted cells, nanopipettes were integrated on a scanning ion conductance microscope (SICM). An SICM integrated electrochemical to syringe is designed for the RNA and organelles extraction (Fig. 6a) [126]. In principle, ion current at the SICM nanopipette tip was kept positive to prevent the aqueous solution entrance when approaching cell. After the nanopipette penetrate the cell, the potential inside the nanopipette will be negative, and the cytosol can be collected. The collected mRNA and organelles can be analyzed by amplifying or sequencing. Furthermore, a double-barrel SICM probe was employed (Fig. 6b) [127], where barrel with aqueous was used for morphological mapping, and barrel with organic solution was used as the electrochemical syringe for the high-resolution imaging.

Semi-implantable devices can be applied for extracellular electrochemical detection by monitoring the redox currents of vesicle content secreted by cells. In one study, carbon-fiber microelectrodes with conical nanotips are employed to detect catecholamine of individual nanoscale vesicles from intracellular microenvironment (Fig. 6c) [128]. The nanotip minimalized the damage to the cell during the intracellular detection. After the penetration, the limiting current drops to a low level, which indicates good sealing between the cell and nanoelectrodes. Besides, the limiting current recovers to 95% of initial one after electrode withdrawal from the cell, implying the good stability after semi-implantable operation. Recently, dielectrophoretic nanotweezers (DENT) were proposed as a powerful tool to extract mRNA from intracellular microenvironment. Using minimally invasive nanotweezers, the precise and spatial sample extraction can be performed from living cells (Fig. 6d) [129]. DENT usually consists of 10–20 nm nanoelectrodes for trapping of DNA, protein, mitochondrion by dielectrophoretic. This attractive DENT provides the precise single-molecule or organelle manipulation to understand the living cells.

Melosh et al. developed a nanostraw-electroporation system defined as nanostraw extraction (NEX) for subcellular content analysis [130]. NEX device is based on a porous polymer membrane with hollow nanostraw array. Indium tin oxide (ITO) substrate and a Pt electrode were assembled for the electroporation (Fig. 6e). After the electrical pulses are applied, the nanopore will appear on the cell membrane, and the intracellular content such as mRNA or protein can be extracted. The main extraction process depends on the diffusion of intracellular content to the external reservoir, while the positive electrical pulses from ITO improve the negative charged contents moving toward the lower reservoir. Based on this nanostraw-extraction strategy, the extraction can be repeated on the same set of cells with high cell viability (> 95%). Moreover, gene analysis in extracted cytoplasmic substance extracted by the NEX platform was analyzed in detail, where 41 mRNA molecules were accurately detected in a quantitative manner. Further, the NEX can also efficiently collect proteins such as lactate dehydrogenase B (LDHB), where the quantitative analysis of extracted LDHB suggested the good repeatability and consistency of NEX for protein extraction and downstream analysis.

#### Electrophysiological Recording

**Fig. 7:**
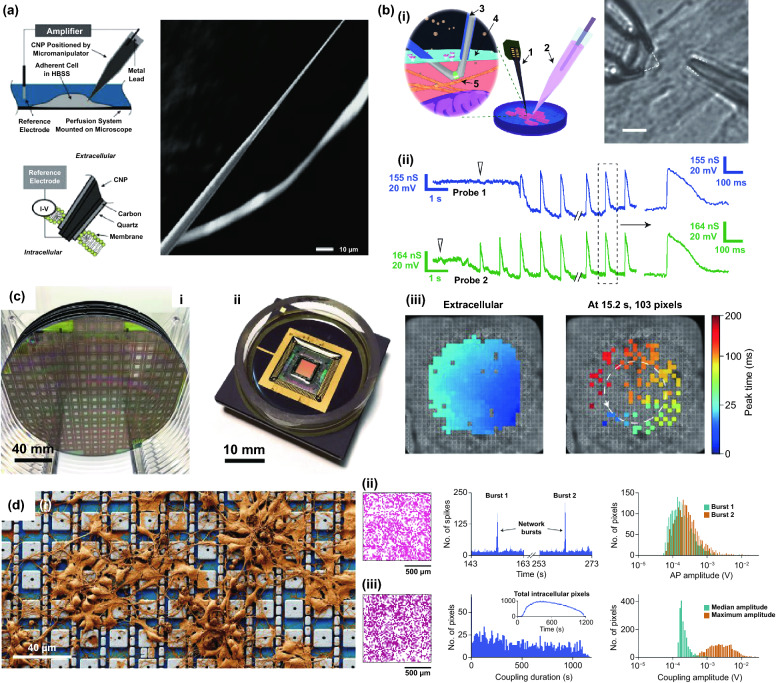
**a** Schematic diagrams of the CNP electrophysiology experimental setup and the interface between CNP and cell membrane (left). SEM image of CNP tip (right) [135]. **b** (i) Left: Schematic diagram of intracellular electrical signal recording from a target cell by independent kinked nanowire transistor probes. Right: A differential interference contrast image of the twisted nanowire probe and the diaphragm clamp pipette. Scale bar: 5 µm. (ii) Multiplexed IC APs were recorded from two adjacent cardiomyocytes using a dual kinked nanowire probe [105]. **c** (i, ii) Images of CMOS fabrication and assembly. Each wafer containing 256 integrated circuits. (iii) Prior to electroporation, extracellular action potential mapping shows uniform signal propagation. After electroporation, the mapping showed a significant decrease in intracellular signal conduction velocity and spiral reentrant behavior [82]. **d** (i) Pseudo-color map of neurons cultured on CNEI arrays. (ii) Intracellular recordings of two bursts on a CNEI array showing synchronized firing. (iii) Another array range of intracellular recordings on cells was made in 19 min of recording by continual stimulation and a total of 1,728 pixels of intracellular coupling [32]. Reproduced with permission from Refs. [32, 82, 105, 135], copyright (2020) Nature Publishing Group (2017) Nature Publishing Group (2014) Nature Publishing Group and (2009) American Chemical Society Publishing Group

Neuroscience and cardiology are both the research focus in biomedical field. In vitro cell models (e.g., neurons, cardiomyocytes) are widely adopted for studies, since in vivo experiments are more inconvenient to perform. Though extracellular electrophysiology is well developed for current neuroscience, intracellular electrophysiology is concerned as more attractive way to explore the activities of neural network or brain [131–134]. Patch clamp, as the gold standard electrophysiological invasive devices, provides recording of high-quality action potential by forming a coupling interface with intracellular environment through suction of cell membrane. However, this invasive working mode is difficult to perform for long-term and high-throughput recording. The glass micropipettes of patch clamp are relatively large for single cells, which induces larger damage to the cells. Multielectrode arrays, a noninvasive device, can chronically record the extracellular signals from multiple cells in network, which possess high cell viability. However, signal quality of extracellular recording is limited due to the weak coupling of electrode with cells, rarely able to reflect the detail information of action potential. Compared with invasive devices and noninvasive devices, semi-implanted electronics could provide intracellular recording with a high-quality and long-term profile, which seldom affect cell viability and became applicable for the recording of vast excitable cells for the neuroscience and cardiology. To overcome these limitations, nanoscale devices have emerged for high-throughput recording of intracellular electrical signals in a minimally invasive manner to the cells. Nanoelectrode platforms could provide intracellular recording with a high-quality and long-term profile, which became applicable for the recording of vast excitable cells for the neuroscience and cardiology. For example, carbon-based nanoelectrodes were developed and utilized by Schrlau et al. for the minimally invasive intracellular recording (Fig. 7a) [135], where carbon nanopipettes (CNPs) were integrated in the pulled glass capillaries to monitor the HT-22 neurons using a patch clamp current amplifier mode. Moreover, CNPs possess multifunctional properties such as intracellular chemical injection and electrical measurement without damage. In addition to passive nanoelectrodes, active nanostructure of 3D FET nanobioprobes were successfully applied as semi-implantable device for intracellular electrophysiological investigation. Based on the previous research of kinked-SiNW [74], free-standing 3D nanoFET was fabricated by Qing et al. (Fig. 7b) [105], which can facilitate the large-scale and precise positioning recording on cardiomyocytes. In contrast to the free-standing patch clamp, the 3D nanoFET can record the action potential from the cardiomyocytes with high consistency. The semi-implantable nanowire device guides a promising direction to establish a biocompatible and high-coupling cell-nanointerface for the investigation of intracellular information.

The pioneering work on nanowires-based semi-implantable devices for excitable cells recording demonstrates the feasibility of high-throughput intracellular recording by patterned nanowire array [90, 101]. Abbott et al. integrated Pt/SiO_2_ nanowire arrays with complementary metal-oxide–semiconductor (CMOS) device for high-resolution and high-throughput intracellular recording for in vitro cells (Fig. 7c) [82]. The nanoelectrodes array worked in pixel unit, which was coupled on amplifier and stimulator modules to record or stimulate the cells. By electroporation of stimulation module, 5 mV amplitude intracellular signals are recorded. Owing to the high-resolution pixel integrated circuit, electrophysiological activities of cell network can be visually monitored. In recent study, intracellular recording of thousands of neurons was performed simultaneously by the same group (Fig. 7d) [32]. The device contains two working mode, the pseudo current-clamp mode and pseudo voltage-clamp mode. In pseudo voltage-clamp mode, the ion channel currents can reflect the drug effects, while intracellular action potentials and postsynaptic potentials of neurons can be recorded in pseudo current-clamp mode. Pixel nanoelectrodes can map the excitatory and inhibitory synaptic activities from a large number of neurons in long-term manner. This high-throughput and high-resolution intracellular recording can provide a unique visualized platform for the large-scale neural networks.

### Limitation and Future Trends for Cellular Applications

Semi-implantable devices can achieve the precise manipulation or sensing in cells by the specialized nanointerface. The nanowire arrays significantly enhance the throughput of single-cell regulation. By various powerful assisting strategies, the efficiency of penetrate can be significantly improved while the cell viability can maintain at a high level. Though these semi-implantable nanowire devices present versatile performance for cell applications, limitations still exist and need to be improved in the future: (I) Reducing nanofabrication difficulty. For nanofabrication, the advanced and complicate instruments should be applied which hampers the low-cost, high-efficiency, and large-scale production. (II) Improving the penetration success ratio. The cell penetration is improved by the semi-implantable platform, yet the ratio is still low. More strategies should be developed for the higher penetration probability. (III) Designing the multifunctional semi-implantable device. In most cases, the semi-implantable nanodevices possess single function, which lack the recording of high-content intracellular synchronous information. With the rapid development of semi-implantable nanodevice, the intracellular research will be more deeply and widely prompted in the near future.

## Semi-implantable Device for In Vivo Applications

### Principle and Strategies

Thousands of complex life activities widely spread in human or animal body, while the understanding of the real-time mechanism of these activities requires detection and analysis in vivo. The advantage of recording biological information in the tissue feature with noninvasion and convenience, yet the recording accuracy and timeliness are compromised due to the barriers of skin or skull. To overcome these barriers, semi-implantable devices that integrated with penetrating probes could serve as promising tool to bypass biological barriers to assess the in vivo tissue. The in vivo semi-implantable devices are generally consisted of the transdermal sensor and external control circuit system for in vivo signal sensing and external interventional modulating. To maximally avoid tissue damage or caused pain of the living animal or human body after probe insertion or implantation, these implanted probes are developed toward miniaturization and flexibility. Moreover, the coating of the probe surface with biocompatible coating materials could reduce the adverse inflammation or fibrosis effects caused by the implanted probes, while the bulk external circuit systems remained on the body surface could be further flexibly designed for practical applications. Dependent on the applications and target tissues, the sensing probes or interventional modules of these functional devices are generally implanted into the transdermal tissue, soft tissue, or brain tissue by assist of the tiny sharp tip-feature of their probes, or by externally assisting metal needles which guide the probes to the target site in vivo. To ensure the effective and safe application in vivo, the designs and developments of semi-implantable devices should take the following issues into account: (1) Minimal invasion; (2) Biocompatibility; (3) Accuracy and sensitivity of detection; (4) Long-term, real-time and in situ applications. Based on the above design principles, the development of in vivo semi-implantable bioelectronic devices have achieved reasonable progress during the past decades, particularly with transdermal devices, microneedles devices and brain electrodes as typical successful examples. For examples, transdermal devices and microneedles devices are designed to penetrate skin layer so that the probes could electrochemically sense or regulate the in vivo tissue environments, which have been emerging as new generation tools for the diagnosis and treatments of diseases such as diabetes. In addition, brain electrodes, which are designed to record or stimulate electrical signals in brain tissues by placement of implanted electrode in the target brain area, have also shown great potentials not only on the treatments of diseases such as Parkinson's disease (PD), Alzheimer's disease (AD), and so on, but also have been demonstrated as powerful tools to monitor and map the electrical activities of brain that could facilitate understanding of brain in nature.

### Devices and Applications

#### Transdermal Device

**Fig. 8:**
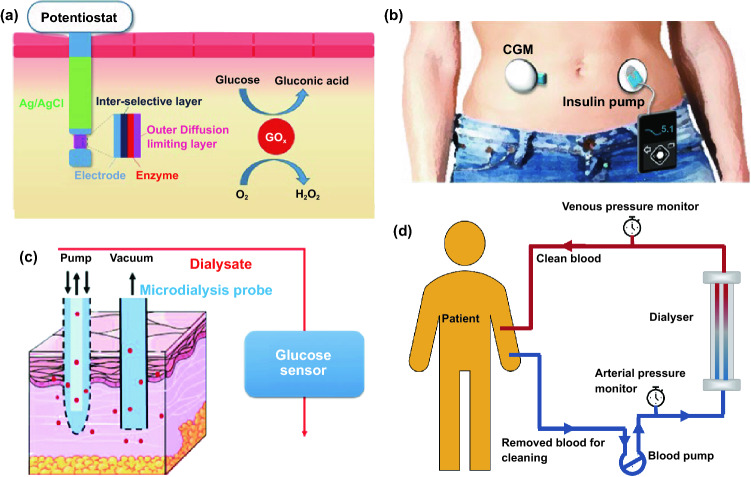
Representative transdermal semi-implantable device. **a** Configuration and principle of percutaneous electrochemical glucose biosensor. **b** CGM-based close-loop insulin pump system. **c** Microdialysis probes for glucose monitoring [152]. **d** Hemodialysis system. Reproduced with permission from Ref. [152], copyright (2015) The Royal Society of Chemistry Publishing Group

For biomedical diagnosis, many indicators in blood reflect the health status. For example, the blood glucose level reflects the health of pancreas, cholesterol and triglycerides level presents the health of cardiovascular, and protein level indicates the health of other organs. Moreover, the metabolism monitoring of drug concentration in vivo is of critical important in clinical therapeutics. A large amount of point-of-care test (POCT)-based blood monitoring has been widely employed in clinical practice. If the blood analysis can be detected in situ, the pain of frequent blood drawing can be relieved. Significantly, in situ detection can achieve the real-time monitoring to understand the dynamic changes of diseases. While biomarkers that are generally rich in blood could directly reflect the health status, the access to blood vessel with external probe or devices possesses undesirable risks of arterial or venous bleeding or infections. Therefore, the applications of implanted sensors that directly access to the blood vessels have not been widely explored. Instead, transdermal device that could access to the tissue in epidermis or dermis has been developed to detect biomarkers in the interstitial fluids, which could somehow reflect the states of biomarkers in the blood vessels. For example, the concentration of glucose in interstitial fluids has been found to be positively correlated with the concentration of glucose in the blood, although the change of glucose concentration in the interstitial fluids existed a 5–10 min-delay compared to the glucose change in blood. In addition, many types of small molecules, such as reactive oxygen species, lactic acids, uric acids, and nitric oxides, have been demonstrated to be detected from interstitial fluids as biomarkers for diseases. Transdermal devices are effective tools to detect or regulate biochemical activities in the subcutaneous tissue, with typical examples of continuous glucose monitoring (CGM), glucose microdialysis probes, CGM-based close-loop insulin delivery system, and hemodialysis circulation system. To date, the CGM based on enzyme-based electrochemical detection of glucose concentrations is one of the most successful technologies of transdermal devices and has been commercialized for clinical applications. CGM as transdermal sensors possesses enzyme-based glucose electrode inserted through the skin to detect glucose concentration in the interstitial fluidic environment. Enzyme-based CGM biosensors are sensitive and selective to glucose due to the specific glucose oxidase (GOx) that could catalyze glucose into hydrogen peroxide production. At present, the CGM technologies of three companies, Dexcom [39], Abbott, and Metronic [136], can achieve continuous measurement of blood glucose changes in ISF [40]. In contrast to self-monitoring of blood glucose (SMBG), CGM can continuously track blood glucose trends in long term. In principle, the glucose detection can be amperometrically performed by measuring the oxygen consumption or hydrogen peroxide production [137, 138]. Under the catalysis by GOx, the glucose was oxidated to gluconic acid which the oxygen was reduced to hydrogen peroxide, so the glucose concentration can be quantified by amperometric signal from the produced hydrogen peroxide (~ + 0.6 V vs Ag/AgCl) or the consumed oxygen (~ -0.6 V vs Ag/AgCl). Based on the specific enzyme catalytic reaction, the electrochemical biosensor is one of the most popular and utilized platform for clinical CGM device (Fig. 8a) [41, 139]. The performance of glucose biosensors is related to electrode design, enzyme coating status, and biocompatible membrane. However, the performance of these biosensors is limited by lack of oxygen or interference of hydrogen peroxide detection by other electroactive endogenous components such as ascorbic acid, uric acid, and acetaminophen [140]. To solve these limitations, anti-biofouling permselective membranes (e.g., Nafion, polycarbonate) are employed to reduce the glucose and electroactive interference diffusion around the enzymatic biosensor, which is effective to relieve the O_2_ deficiency and electroactive interferences [141–144]. To eliminate the O_2_ deficiency, the glucose dehydrogenases (GDHs) was employed in glucose sensors, which can work without O_2_ supply with various cofactors, such as flavin adenine dinucleotide (FAD), pyrroloquinoline quinone (PQQ), or nicotinamide adenine dinucleotide (phosphate) [NAD(P)] [145, 146]. Though the GDH is independent of O_2_ concentration, the FAD/PQQ-GDH can overestimate the glucose concentration due to catalyzing the other biomarkers such as maltose, while the oxidization of NAD(P)H produces other polymerized oxidation products to foul the electrode and increase the overpotential [147–151].

Since the glucose concentration in ISF is correlated with that in blood, the ISF glucose could be continuously monitored by the transdermal probe [152–155]. The implanted electrodes-type sensors have been widely employed to accurately reflect the blood glucose level in real time due to their advantages of timeliness and portability as wearable medical system. The in vivo continuous blood glucose monitoring was first proposed in 1982 [156], and the implanted electrodes-based CGM system was approved and commercialized by Food and Drug Administration (FDA) in 1999 [157]. Though ISF-based CGM system still lacks accuracy compared with the blood glucose meter, they have been successfully demonstrated to achieve the glycemic control and reduce the hypoglycemic events [158–160]. Most of the commercially available CGMs transdermally measure ISF glucose to reflect the blood glucose level in a given interval of 5–15 min. The transdermal electrode is inserted into a defined area of human body by the assistance of metal needles pushed by a mechanical device. The current change on electrodes in response to glucose levels is received by the external sensor attached on skin, and the CGM transmitter sends the glycemic data to receiver by wireless communication. Due to the blood glucose dynamic balance between the vessel and ISF, the calibration algorithm is established based on the plenty of clinical data from CGM to calibrate the blood glucose delay of ISF. Generally, the blood glucose delay in ISF is 5–10 min. Compared with intermittent capillary blood glucose measurement, CGM can perform the continuous glycemic measurements using semi-implantable enzyme-tipped electrodes, and these transdermal sensors can stay in vivo for 1–2 weeks before taking them out for calibration [161–164].

However, issues of system reliability, noise interference, and frequent calibrations hinder the marketing, until the new Libre CGM system emerges to be approved by FDA [165–168]. Traditional sensors of CGM generate the large noise during 1–3 days of initial implantation, while the reasons are still unclarified [168–170]. Consequently, the FDA had approved traditional CGMs can be employed for 1 to 2 weeks after implantation, while SMBG (finger-prick blood test strip) should be applied for frequent recalibrations (i.e., 4 time on initial day and once every 12 h later) [165, 171]. The inaccuracy from noise issue prolongs around 30% to 50% approved period of CGM products, while the frequent recalibration operations lower humanization and are painful for the users, resulting in the unreliable blood glucose measurements [172–174]. To reduce the noise and improve the accuracy, the various materials are used as an antifouling coating on glucose sensors. Hu et al. polymerized zwitterionic sulfobetaine methacrylate monomers on the GOx-coated sensor with bromination. It is demonstrated that the antifouling coating can diminish 99% nonspecific protein adsorption, maintain long-term high sensitivity, and improve the inaccuracy, compared to the commercial glucose sensors [175]. Another antifouling polymer-coated glucose sensor was fabricated by a zwitterionic poly(sulfobetaine-3,4-ethylenedioxythiophene) (PSBEDOT) by one-step electropolymerization. By this antifouling polymer coating, the sensor presents a high linearity (*R*^2^ = 0.9874) from 0.1 to 0.5 mM. In contrast to antifouling properties of PEDOT–GOx coating, PSBEDOT–GOx showed better antifouling properties for blood plasma and fibrinogen proteins [176].

In recent work, Xie et al. developed a high-performance poly(MPC) from 64 types of zwitterionic polymers by combinatorial chemical approaches as an antifouling coating on the Medtronic CGM to relieve the inflammation and potential signal noise [177]. Using the biocompatible polymer, the CGM performance was significantly improved with lower signal noise. To verify the practical applications, the polymer-coated sensors were assessed by mice and non-human primates, and the sensors can measure the accurate blood glucose without recalibration. Moreover, the immune responses were proved to be inhibited by this polymer by histology and inflammation-associated protease, and gene expression of inflammation biomarkers. Significantly, the polymer coating will be promising approach to enable CGM as a standalone device. In addition to continuously record glucose fluctuation in real time, the regulation of glucose levels in vivo could be achieved by the insulin pump. Insulin pump is an intelligent system that biomimics the secretion of human pancreas. Via the artificial intelligence control, the insulin pump simulates the regulation of basic insulin in the body by a tunable pulse subcutaneous infusion. Insulin pump system typically includes artificial intelligence control system with microelectronic chips, battery-powered mechanical pump system, drug reservoir, connected infusion tubings, and subcutaneous infusion catheter. One end of the infusion tube can be implanted under the skin of the patient. In the operating state, the pump mechanical system receives commands from the control system to drive the piston of reservoir, which will eventually work as a pancreas to provide the insulin.

The crucial significance of CGM function is associated with insulin pump to be a smart system. To automatically and accurately regulate the daily blood glucose of diabetics, CGM-based close-loop insulin pump is established as an artificial pancreas (Fig. 8b), which is an advanced in vivo medical system with a semi-implantable device for routine glycemic regulation of diabetics [178–182]. CGM works as a sensor during the glycemic monitoring, while insulin pump serves as an actuator. Self-inserted Teflon or steel catheter is connected with the insulin reservoir of pump by a long tubing. With the development of manufacturing technology, the volume of CGM-based close-loop insulin pump system has been dramatically reduced and facilitates to carry, learn, and operate, where the dose adjustment is more accurate and stable. Consequently, it has been widely applied in clinical practice. At present, the technology of insulin pump is more advanced in that it can precisely simulate the physiological secretion of insulin. Briefly, the insulin pump can be regulated by artificial intelligence to simulate the basal insulin secretion in the body by an tunable pulsed subcutaneous infusion. Generally, the closed-loop insulin system usually delivers the insulin directed by a control algorithm according to the real-time ISF glucose concentration from matched CGM system [161, 178, 183].

In addition to enzymatic electrochemical glucose biosensor, semi-implantable microdialysis technique is an alternative way to collect the dialysate from blood [184–186] or ISF [187–189], which could be further analyzed by an external glucose sensor (Fig. 8c). The microdialysis probe is coated with a semipermeable membrane and inserted into the tissue, and the glucose in collected dialysate could continuously perfuse to the measuring module of glucose sensor. Compared with the ISF microdialysis, the intravenous microdialysis possesses the advantages that can accurately measure glucose [190] directly from the blood, yet the intravenous nature is more invasive. Microdialysis technique has a readout lag (~ 5 min) due to time-consuming dialysate transportation to the glucose sensor. While microdialysis could potentially enable multiplexed detections by directly extracting fluids compared to implanted electrodes, the various peripheral devices (e.g., pump, tubing, and sensor) more significantly affect the physical activities of users than form of implanted electrodes, which limits its applications to the clinical diagnosis [191–194]. In addition, though microdialysis-based glucose derives the ISF or blood sample to measure the glucose concentration ex vivo, this method is unstable due to the foreign body response (FBR) with the disadvantages of longer analytical time for glucose measuring [140, 195, 196].

In addition to the CGM-insulin pump system that mimics the natural pancreas, hemodialysis monitor is another widely used transdermal device system that could mimic the kidney to remove the waste and purify the blood [197–199]. By this way, the hemodialysis system could regulate the blood physiological environment. When the transdermal tubings are fixed on the patient’s body (Fig. 8d), the blood is continuously treated in the hemodialysis machine consisting of key water system and dialyzer. The water system mainly contains dialysate and heparin pump to refresh the blood and prevent clotting, while the dialyzer is employed to filter the creatinine, urea and water from the blood. For the safety consideration, the newest dialysis machines are continuously monitor an group of safety–related parameters, such as blood and dialysate flow rate, blood pressure, dialysis solution conductivity, pH, and temperature to eliminate the potential risk of blood leakage or air formation.

#### Microneedle Device

Commercial medical transdermal devices (e.g., CGM, glucose microdialysis probes, close-loop insulin delivery system, and hemodialysis circulation system) can efficiently achieve the sensing, delivery, or sampling from the in vivo environment. However, the large needles or implanted probes induce uncomfortable experience or potential medical risk due to the nerve or blood contact. Microneedles technology has emerged as a novel form of transdermal devices with 500–800 μm-length needles in an array, which could subcutaneously penetrate skin layer in a painless and minimally invasive way. The short microneedles were intentionally intended to penetrate the stratum corneum, which is the outermost layer of the skin, but without reaching to the nerve endings or blood capillaries in the dermis layer, enabling penetration of skin in a painless manner. Moreover, the microneedle technique reduces the operational complexity of well-trained medical personnel, which makes it a convenient tool for non-professional personnel. Furthermore, the minimal invasive and in situ feature effectively avoids the blood extraction and reduces the possibility of undesired problems as blood infections, sample contamination and so on, which pave a convenient and alternative path for transdermal applications. Combined with the well-established portable detection or delivering system, the detection tasks could be performed by the patients without the concerns or risks of tissue damages or infections caused by metal needles.

**Fig. 9:**
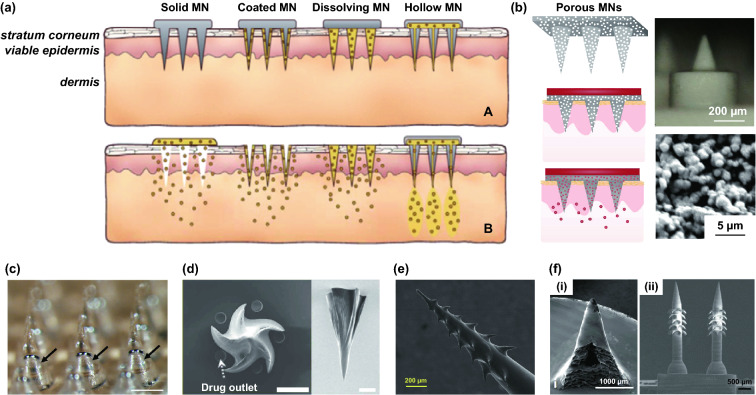
**a** Transdermal drug delivery strategies using microneedles: (A) Various types of microneedles apply to the skin, and (B) corresponding methods used for drug delivery [200]. **b** Drug delivery strategy (left column) and SEM images (right column) of porous microneedles [201, 202]. **c–f** Micrographs of representative novel physical forms of microneedles, including **c** rapidly separable microneedles (scale bar: 500 μm) [204]. **d** grooved microneedles (left: top view, right: lateral view. scale bar: 100 μm) [205]. **e** magnetorheological lithographed microneedles [209]. **f** 3D printed microneedles with internal hollow (i, [7, 212]) and with backward-facing barbs (ii, [12, 213]). Reproduced with permission from Refs. [200–202, 204, 205, 209, 212, 213], copyright (2012) Elsevier B.V Publishing Group (2021) Elsevier B.V Publishing Group (2021) Nature Publishing Group, (2019) Nature Publishing Group, (2019) American Association for the Advancement of Science Publishing Group, (2018) American Chemical Society Publishing Group (2020) Wiley-VCH Publishing Group and (2020) Wiley-VCH Publishing Group

Transdermal deliveries of drugs, vaccines or diagnostic agents are important application of microneedle technique in the past decade. The strategies of medicine deliveries are generally determined by the physical forms of microneedles. Traditional forms of delivery-purpose microneedles are summarized in Fig. 9a [200]. Solid microneedles can be used to create micron-scale pores in the skin surface, following by drug formulations applied to the skin or tip-coated drugs remaining in the skin for slow diffusion. Water-soluble or swellable microneedles encapsulate the medicines within the tips, thereby releasing them slowly along with the tips dissolving or degrading in the skin. Hollow microneedles can be used for delivery liquid formulations in precise dose. In recent years, emerging porous microneedles were proved to be another solution for transdermal controlled release, with relatively facile fabrication, as shown in Fig. 9b [201–203]. Specific physical forms were developed for unique applications as well, such as integrating soluble microneedle tips and bubble structure into a separatable microneedles (Fig. 9c) [204] and grooved microneedles inspired by snake fangs (Fig. 9d) [205] for efficient transdermal and liquid-formulations delivery.

In addition to traditional photolithography and etching methods, researchers have developed a number of strategies to fabricate and optimize the structures of microneedles, including hot embossing, magnetorheological lithography (Fig. 9e) and 3D printing (Fig. 9f). Hot embossing is a common method for fabricating microstructures into shapes, which can make solid microneedles, is low-cost and easy to handle, but has high requirements for molds [206, 207]. Magnetorheological lithography can effectively fabricate microneedles without the use of molds, which is efficient and more flexible, but has limited material options [208, 209]. 3D printing is a relatively new fabrication with high design flexibility, high efficiency, and greatly reduced manufacturing difficulty, but also has the disadvantage of limited material options [210–213].

**Fig. 10:**
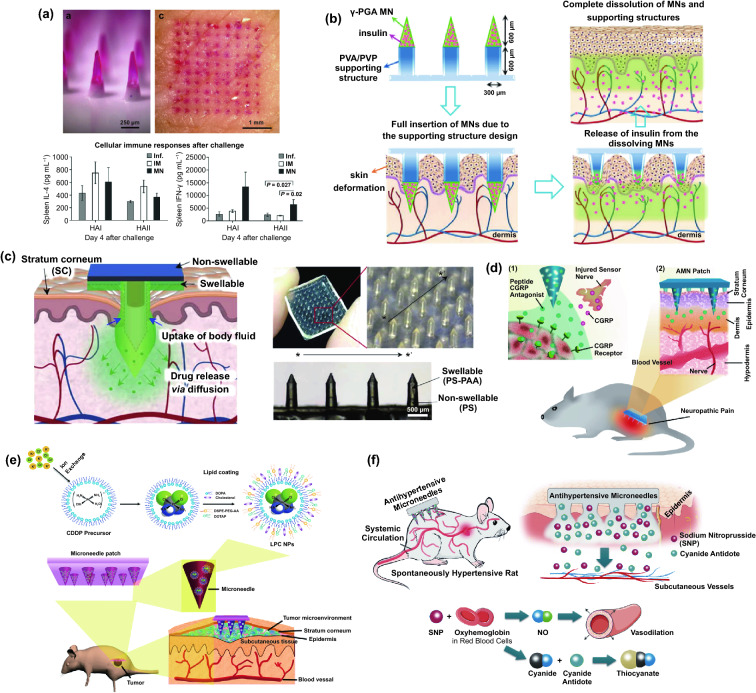
Water-soluble or biodegradable microneedles used for hypodermic formation delivery. **a** Dissolvable microneedle delivered inactivated influenza virus and achieved effective vaccinations compared with traditional intramuscular immune (i.m.) immunization [214]. **b** Schematic diagram of insulin delivery by microneedle composed of poly γ -glutamate (γ-PGA) and PVA/PVP [216]. **c** Swellable microneedles used for prolonged release of insulin [218]. **d** Longer hypoglycemic effect than subcutaneous injection demonstrated in diabetic rats, using gelatin composites microneedles for slow drug releasing [219, 220]. **e** Schematics of delivering CGRP antagonist peptide for curing localized neuropathic pain via dissolvable MNs [221]. **f** Cisplatin loaded microneedles were employed for safe and efficient antitumor therapy [222]. **g** Biodegradable microneedle used for combinative delivery of SNP and sodium thiosulfate via biodegradable microneedles for side effects controlled antihypertensive therapy [223]. Reproduced with permission from Refs. [214, 216, 218–223], copyright (2010) Nature Publishing Group, (2018) Elsevier B.V. Publishing Group, (2017) Elsevier B.V. Publishing Group, (2017) Elsevier B.V. Publishing Group, (2017) Elsevier B.V. Publishing Group, The Royal Society of Chemistry Publishing Group, (2017) American Chemical Society Publishing Group, (2018) American Chemical Society Publishing Group and (2019) American Chemical Society Publishing Group

In recent years, water-soluble or biodegradable microneedles became the most studied category due to the convenience in preparation and the versatility in treating diseases. Sullivan et al. developed dissolving microneedle patches encapsulating inactivated influenza virus vaccine that targeting delivery to skin’s antigen-presenting cells, providing facilitated vaccination and improved immunogenicity on mice model compared to conventional intramuscular injection (Fig. 10a) [214]. Wang et al. introduced nonablative fractional laser (NAFL) treatment on local skin before inserting the influenza vaccine-packaged, biodegradable microneedles, achieving lesion-free cutaneous vaccination and broadened cross-protective immunity owing to the NAFL-mediated adjuvanticity [215]. Subcutaneous insulin delivery via biodegradable microneedles is another hotspot (Fig. 10b) [216, 217] as they can provide the essential continuous delivery for protein drugs. For instance, Seong et al. developed swellable PS-PAA microneedles with high percentage of effective insulin dose loaded in the swollen polymer network, leading to prolonged release insulin rather than a burst release (Fig. 10c) [218]. Moreover, Yu and co-workers designed and fabricated gelatin/calcium sulfate and gelatin/hydroxyapatite composite microneedles for insulin delivery, which presented longer hypoglycemic effect than subcutaneous injection route in diabetic rats model (Fig. 10d) [219, 220]. Localized therapy for avoiding systemic side effects is another advantage of microneedle-based drug delivery. Xie et al. utilized dissolvable microneedle to transdermally deliver selective CGRP antagonist peptide for curing localized neuropathic pain on rats model in a painless and irritation-free manner, without disturbing the normal nociception and motor function (Fig. 10e) [221]. This advantage also provides a promising dosage form for antineoplastic drugs with systemic toxicity and side effects. Su’s team reported a safe subcutaneous delivery of lipid-coated cisplatin nanoparticles via dissolving microneedles, resulting in remarkable reduction in tumor volume and weight within a xenograft tumor animal model, and non-organ toxicity was detected in the meantime (Fig. 10f) [222]. On the other hand, capillaries in subcutaneous tissues can allow fast diffusion of small-molecule drugs from the dermis into the systemic circulation, offering new opportunities for self-administrable cardiovascular diseases therapeutics. Li and co-workers successfully developed biodegradable microneedles for combinative delivery of sodium nitroprusside (SNP) for treating hypertensive emergencies and sodium thiosulfate for suppressing side effects induced by SNP, on the spontaneous hypertensive model (Fig. 10g) [223].

**Fig. 11:**
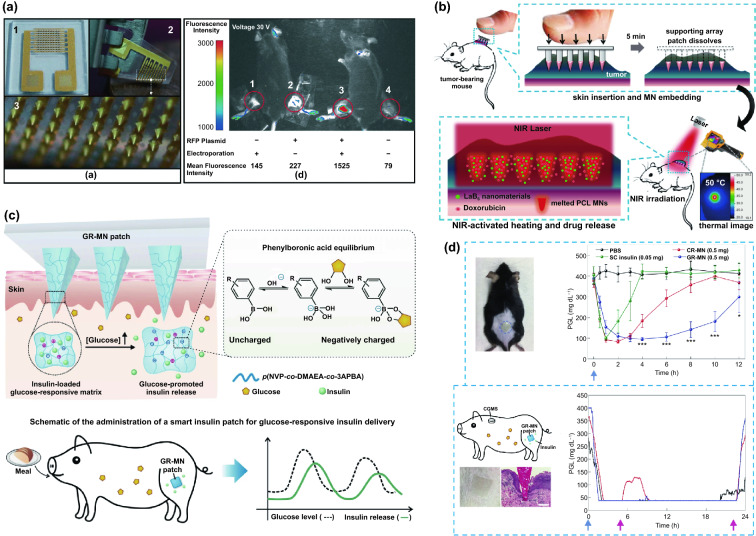
Microneedle-based therapies with on-demand drug release. **a** Left: conductive microneedle array interdigitated electrode for electroporation. Right: Microneedle electroporation treatment effectively enhanced the expression of pmRFP-C1 (RFP) plasmid (area 3) [224]. **b** Schematics of near-infrared (NIR)-light-triggered transdermal controlled-release microneedles for chemotherapy and photothermal treatment of tumor on mice model [228]. **c** Illustrations of glucose-triggered insulin release from glucose-responsive microneedle (GR-MNs) and the application of GR-MNs on diabetic minipig model [230]. **d** Effective and rapid regulation of plasma glucose levels (PGL) in insulin-deficient diabetic mice (upper) and minipigs (bottom). Blue arrows: time points of microneedle administration. Pink arrows: time points of feeding [230]. Reproduced with permission from Refs. [224, 228, 230], copyright (2014) The Royal Society of Chemistry, (2016) American Chemical Society Publishing Group and (2020) Nature Publishing Group

Based on micro-nano fabrication technology, microneedles are feasible to combine with other advanced technologies for achieving more complex functionalities, e.g., on-demand drug release. Wei et al. integrated conductive microneedles electrodes with electroporation device for in vivo DNA and siRNA delivery under safe voltage, achieving efficient and localized delivery of plasmid DNA (in healthy muscle tissue) and siRNA (into tumor) in mice model (Fig. 11a) [224]. Chen’s team encapsulated photothermal conversion nanomaterial (LaB_6_) and chemotherapy drug molecules in polycaprolactone microneedles with low melting point (~ 60 °C) to establish a near-infrared (NIR)-light-triggered transdermal controlled-release system [225–227]. Benefitting from the simultaneous photothermal therapy and chemotherapy to superficial tumors, this microneedle system demonstrated good synergistic effect that eradicated 4T1 tumors within 1 week on mice model, without recurrence and significant weight loss (Fig. 11b) [228]. Gu’s team integrated carefully designed glucose-responsive vesicles or matrix with insulin loaded into swellable microneedles for establishing closed-loop insulin delivery systems [229, 230]. The essential glucose-sensing composites such as glucose oxidase and phenylboronic acid can sensitively respond to hyperglycemic conditions and lead to the dissociation of glucose-responsive components, then releasing insulin into systemic circulation via subcutaneous vascular and lymph capillary networks. These systems demonstrated the effectiveness of blood glucose regulation on both insulin-deficient diabetic mice and minipigs models (Fig. 11c, d).

**Fig. 12:**
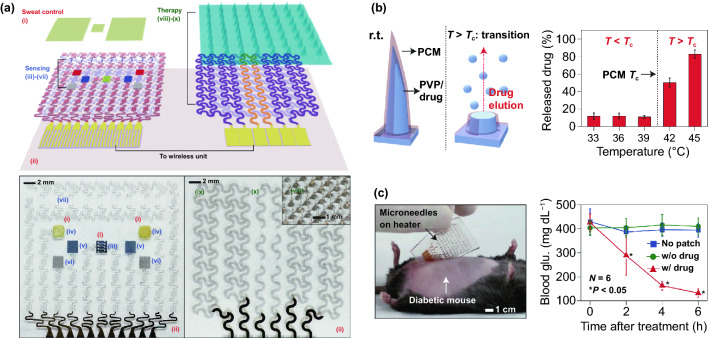
Integrated diagnosis & treatment system using microneedles for controlled drug delivery. **a** Schematics and corresponding photographs of the stretchable skin-mounted closed-loop diabetes monitoring and therapy device, including the sweat glucose-sensing module (left) and electro-thermal triggered therapeutic microneedle patch (right), with GP-hybrid materials widely used. The controlled release mechanism of thermal responsive microneedles (left) and its temperature dependence were illustrated in **b**. **c** By delivering metformin to diabetic mice in vivo, this microneedles treatment presented significant blood glucose suppression [231]. Reproduced with permission from Ref. [231], copyright (2016) Nature Publishing Group

Being as a powerful transdermal drug delivery platform, microneedles technique provided exciting novel possibilities for the development of integrated diagnosis & treatment system, by combining with wearable biosensing system. Representative research on closed-loop diabetes monitoring and therapy was reported by Kim’s team [231]. The stretchable skin-mounted system presented in Fig. 12a was mainly composed of a multi-layer sweat-based glucose-sensing patch and an electro-thermal triggered therapeutic microneedle patch. The glucose-sensing module includes a core electrochemical glucose sensor and other humidity, temperature, pH and strain sensors for glucose level correction and hypoglycemic states prediction, all based on graphene (GP)-hybrid materials. For delivery metformin into ISF, the microneedles with dissolvable PVP body and phase-change material (PCM) coating were warmed by the electro-resistive heater, leading to the melting of PCM (transition temperature 41–42 °C) and the dissolving of PVP, thereby releasing loaded drugs. On the diabetic mice model, the measured over-threshold sweat glucose level triggered the heating of microneedle patch to regulate the blood glucose, forming a feedback-controlled drug delivery system. In their later work, a similar but upgraded system with more efficient sweat glucose monitoring and two-stage metformin controlled release was developed [232]. The innovative microneedles were made by dissolvable hyaluronic acid hydrogel coated with PCM, and loaded metformin was encapsulated in two types of phase-change nanoparticles (PCN1 and PCN2, melting transition temperature at 38 and 43 °C, respectively) (Fig. 12b). This realized precise and multistage drug release in response to the monitored glucose level.

On the other hand, microneedles themselves can be developed in to wearable biosensing devices. In fact, microneedle-based biosensing technique has achieved optimistic progress in recent years. Fabricating microneedles from conductive or semi-conductive materials as transdermal sensing electrodes matrix, and performing sensing via three-electrode electrochemistry or bioimpedance measurement, is the fundamental principles of microneedle-based biosensing (Fig. 12c). Typically, to guarantee the sensing specificity, microneedle tips are modified with certain enzymes or antibodies for targeting the analytes. Similarly, micro-nano-materials/structures are widely used at the tips for enhancing the specific surface area and surface conductance, thereby enhancing the sensitivity of microneedle electrodes. Aside from solid microneedles, hollow microneedles can also provide a technical strategy for biosensing, that utilize micro-channels inside microneedle tips to transfer interstitial fluid (ISF) from the dermis or the endothelium for in vitro analyzing or on-device monitoring via fully integrated microneedle biosensors.

The strength of microneedle biosensing is its minimal invasiveness and convenience in in situ monitoring of subcutaneous bio-signals, while the submicron scale microneedle tips are a double-edged sword that limited its application. First, processing, modifying and integrating tiny microneedles into biosensors challenge the preparation technique, especially for quantity production required by practical application. Second, small microneedle electrodes imply limited surface area that results in weak sensing signals, which typically requires integrating micro-nano-materials/structures at microneedle tips to enhance the specific surface area. It leads to complicated fabricating process and fragile surface structures of microneedle electrodes. Third, the length of microneedle electrodes confines the sensing depth to dermis, lacking the detecting ability in deeper tissue or inside blood vessels at present.

**Fig. 13:**
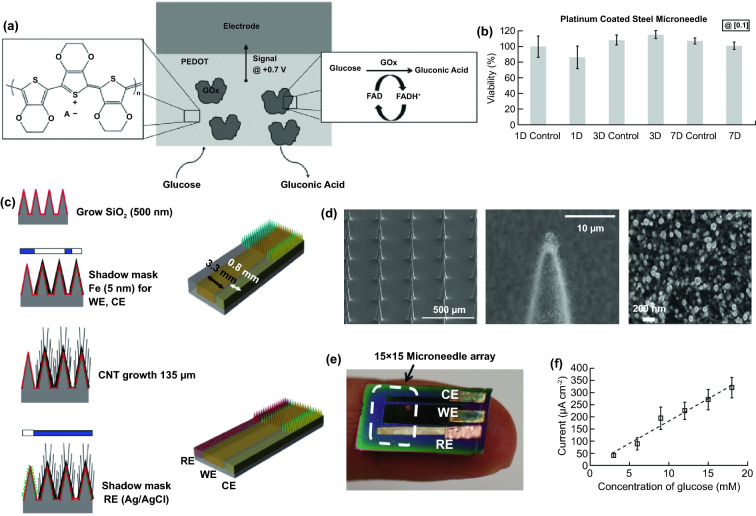
Microneedle-based biosensors for glucose monitoring. **a** Schematic image of glucose-Sensing. **b** Representative concentration curves of platinum-coated steel microneedle sensors [236]. **c–f** An enzyme-free glucose electrochemical sensor with WE, RE, and CE fabricated on an entire microneedle patch. **c** Fabrication sequence. **d** SEM images of microneedle arrays (left), the microneedle tip with MWCNT grown on (middle), and Pt nanoparticles electrodeposited in MWCNT (right). **e** The fabricated 3-electrode microneedle glucose sensor. **f** Current response of the sensor as a function of glucose concentration in PBS [237]. Reproduced with permission from Refs. [236, 237], copyright (2014) John Wiley and Sons Publishing Group and (2013) Multidisciplinary Digital Publishing Institute Publishing Group

Although there are multiple limitations, microneedle-based biosensing is still well suited for continuous physiological signals monitoring and in vivo biomarkers detecting, particularly in continuous glucose monitoring (CGM) [233–235]. Instead of pain and inconvenience caused by frequent finger-stick measurements, or inflammation risks induced by long metallic probe (several millimeters) of commercial CGM devices, microneedle CGM sensors can provide noninvasive or minimally invasive experience for diabetic patients. Invernale et al. demonstrated a representative design of electrochemical working electrode for glucose sensing, utilizing conductive polymer PEDOT to immobilize glucose-specific enzyme GOx on Pt coated stainless steel microneedles (Fig. 13a, b) [236]. High linearity that almost covered the most physiological glucose range of diabetic patients (0–432 mg dL^−1^) and good biosafety were demonstrated in vitro. Another more integrated glucose sensor was developed based on silicon microneedles by Yoon et al.[237]. An entire piece of microneedles arrays was functional zoned into three subareas: the working electrode (WE), the counter electrode (CE) and the reference electrode (RE), via shadow-mask-assisted sputtering (Fig. 13c–f). Iron catalyst, multiwalled carbon nanotubes (MWCNT), and Pt nanoparticle were subsequently modified on the WE and the CE, resulting in good linearity and sensitivity (17.73 ± 3 µA mM^−1^ cm^−2^) as an enzyme-free glucose sensor. Besides the direct electrochemical sensing by modified microneedle electrodes, Jina and co-workers developed a hollow-silicon-microneedle-arrays-based CGM system prototype [238, 239] and achieved good accuracy (overall mean absolute relative difference of 15%) from ten patients with insulin-dependent diabetes for up to 72 h [239]. The glucose in ISF diffused via microneedles’ lumens into the PBS filled sensing chamber, where electrochemical module with GOx-coated working electrode functioned.

**Fig. 14:**
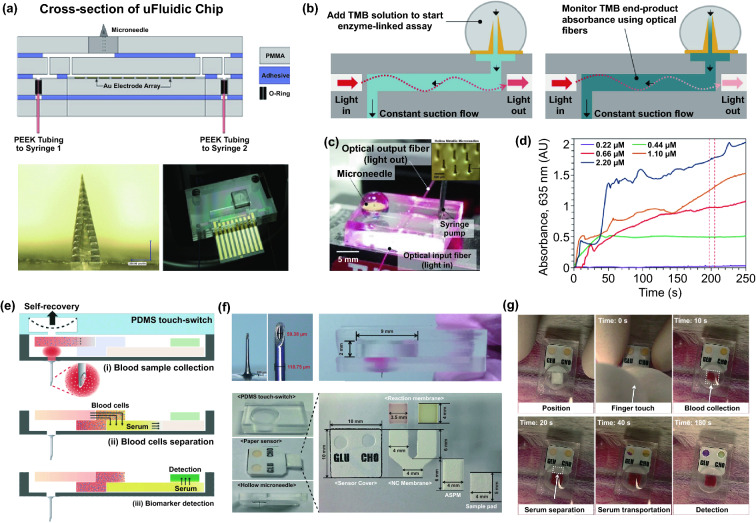
Microneedle-based biosensors for proteins and serum analytes detection. **a** Hollow microneedles and fluidic channels integrated biosensor developed for myoglobin/troponin detection in ISF [240]. **b–d** Hollow-microneedle-optofluidic biosensor for rapid in vitro vancomycin (VAN) sensing [241]. **b** Cross-sectional schematics of the device and the mechanism of optofluidic analyzing. **c** Image of integrated device while analyzing. Inset: the micrograph of the hollow microneedles. **d** Absorbance spectra of different concentration of analytes binding to the microneedle-lumen. **e, f** A fully integrated, one-touch-activated blood multidiagnostic system based on hollow microneedle and paper-based sensors [242]. **e** Mechanism and operating process of the OBMS. **f** Components of the OBMS. **g** Application of the OBMS in the diagnosis of glucose and cholesterol in vivo in rabbit ear arteries. Reproduced with permission from Refs. [240–242], copyright (2016) Wiley-VCH Publishing Group, (2016) Nature Publishing Group and (2015) The Royal Society of Chemistry Publishing Group

A similar hollow microneedle biosensing proof-of-concept demonstration was developed for protein detecting by Miller et al. [240]. Hollow microneedles fabricated by two photon polymerization were integrated with fluidic channels and electrochemical electrodes arrays (Fig. 14a), targeting for myoglobin/troponin detection in ISF. However, this ISF transfer strategy leads to relative long sensing lag time (several min to more than 10 min), which confines its application in detecting large molecules that diffuse slow or therapeutic serum levels that vary fast. Ranamukhaarachchi et al. firstly integrated a hollow-microneedle-optofluidic biosensor for rapid in vitro vancomycin (VAN) sensing [241]. The microneedle lumen immobilized with high-density peptides for VAN recognition acting as the reactive chamber led to low sample volume needed (0.6 nL) and fast responding (< 5 min), while the integrated optofluidic module provided high sensitivity (0.41 AU/decade) and low LoD (84 nM) for VAN quantifying (Fig. 14b–d). Unfortunately, these prototypes are not fully integrated since the necessary modules including ICs and power supplies remain as great engineering challenges to volume and cost control. One clever solution is to directly collect serum samples from superficial vessels via relative long hollow microneedle for non-electronic analyzing (Fig. 14e–f) [242]. By applying the one-touch-activated blood multidiagnostic system (OBMS) on the superficial vessels, approximately 30 μL blood could be extracted into the sample chamber through biocompatible ultra-sharp nickel microneedle [242, 243]. Blood cells were then filtered when the blood flew through the polysulfone membrane. The remaining serum diffused to reaction zones for colorimetric assay. This system was applied on a rabbit ear artery and successfully measured the serum levels of glucose and cholesterol level within 3 min. It provided a powerful platform for diagnosing various biomarker by simply redesigning the paper-based sensor. Similar strategy was employed on glucose detection in ISF [244].

**Fig. 15:**
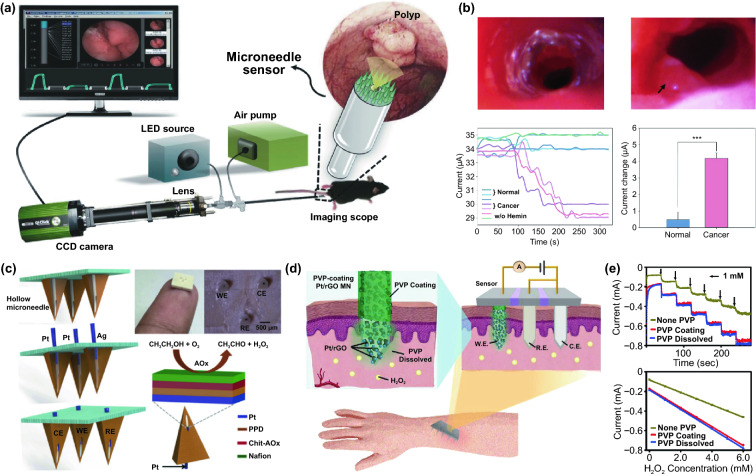
Microneedles-based biosensors for essential physiological biomarkers detection. **a-b** Microneedle sensor and endomicroscopy device integrated dual-diagnostic system for real-time cancer-specific NO signal detection [247]. **a** Schematics of applying the system for in vivo imaging and sensing on colorectal cancer model mice. **b** Real-time in vivo endomicroscopic imaging of normal and polyp regions (black arrow indicates) in mouse colons, and corresponding detected NO signals. **c** Illustrations and images of functionized wire electrodes and hollow microneedles assembled biosensor for monitoring alcohol in ISF [248]. **d, e** Microneedle electrochemical platform with rGO/Pt nanoparticles immobilized and dissolvable polymer coating developed for real-time subcutaneous ROS monitoring [249]. **d** Schematics of the microneedle ROS sensor and the surface structure of WE. **e** Amperometric responses of PVP-coated sensor after inserting into the pigskin and withdraw, referring to the non-PVP-coated sensor. Reproduced with permission from Refs. [247–249], copyright (2015) Wiley–VCH Publishing Group, (2017) Elsevier B.V. Publishing Group and (2019) Wiley–VCH Publishing Group

Apart from the glucose and protein sensing, researchers start to employ hypodermic-microneedle-based approaches for other important physiological biomarkers (pH, K^+^, NO, ROS, alcohol, etc.) monitoring instead of conventional routines. For instance, Miller and co-workers modified the myoglobin/troponin sensing setup (Fig. 15a) into a potassium-ions (K^+^) monitoring platform, by utilizing pyrolyzed carbon as ion selective electrode (the WE) for K^+^ detecting [245]. More recently, an all-solid-state potentiometric microneedles patch for potassium-selective detection was reported by Parrilla et al. [246] Multi-layer coating including potassium-selective membrane-modified microneedle WE directly monitored the K^+^ concentration variation in chicken or porcine skin ex vivo. Scientists also incorporate microneedle biosensors with medical imaging instrument into a powerful dual-diagnostic system [247]. As presented in Fig. 15b, a, hemin/PEDOT/polydopamine-modified PCL microneedle-based electrochemical sensor for highly sensitive NO detecting was mounted on the probe of an endomicroscope, and the probe was applied into the colon of the mice in vivo. Optical images of polyp regions and distinctive increase in cancer-specific NO signal were obtained simultaneously. Alcohol monitoring from ISF is also reported recently by Wang’s team [248]. Alcohol oxidase immobilized Pt wire, Pt and Ag wire electrodes were inserted into the lumens of the hollow microneedles for electrochemical sensing (Fig. 15c), providing a convenient strategy for functioned microneedle electrodes fabrication. Ex vivo mice skin model analysis demonstrated the efficaciousness of this transdermal alcohol monitor. In addition, to protect the fragile micro-nano-sensing structures on microneedle electrodes from mechanical damage in transdermal process, Xie’s team developed a strategy by spray coating dissolvable polymer (PVP) on the microneedle electrodes for in vivo biosensing of reactive oxygen species (ROS) [249, 250]. In this electrochemical sensing platform, PVP protective layer was coated on the microneedle WE that fragile rGO/Pt nanoparticles composites deposited. It provided sufficient mechanical strength to protect the nanostructures on microneedles from damage and dissolved rapidly (< 5 min) in IFS, thereby expose the microneedle surface to function correctly (Fig. 15d, e).

Although fully integrated microneedle-based biosensing and transdermal formation releasing system for in vivo diagnosis and treatment is still being developed, we can expect its appearance and clinical application in the coming future with optimism [235, 251, 252].

#### Brain Electrodes

**Fig. 16:**
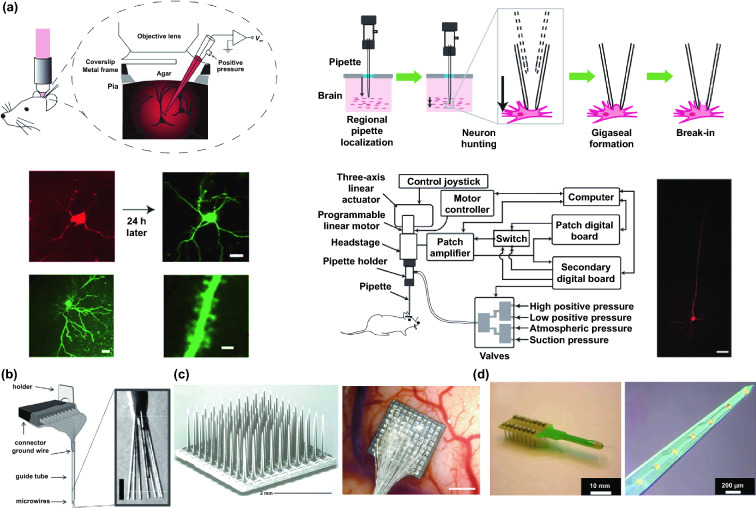
Versatile brain electrodes for neuroscience study. **a** In vivo patch-clamp and automatic patch-clamp for neuronal action potential recording and plasmid DNA delivery electroporation [261, 262]. **b** Traditional microwires and stacked microwires [267]. **c** 100 microelectrodes of Utah electrode array [268]. **d** Single-shank and multiple-shank Michigan electrode array [269]. Reproduced with permission from Refs. [261, 262, 267–269], copyright (2008) Nature Publishing Group, (2012) Nature Publishing Group, (2011) Elsevier B.V. Publishing Group, (2014) Frontiersin Publishing Group and (2008) Elsevier B.V. Publishing Group

Neuroscience becomes the recent research hotspot that mainly reveals the biophysical mechanism in brain, and electrophysiological study is performed to bridge the relationship between physiological activities and electrical responses of neurons. Understanding how single neurons communicate and contribute with each other in the large neuron network is still a big challenge in neuroscience [253–255]. Conventionally, the action potential or ion channel current of signal neuron recorded by single electrode (e.g., patch-clamp) is the gold standard to explore the neurophysiology [256–260]. The patch-clamp technique forms a high impedance seal between glass micropipette and cell membrane by vacuum. Neuron and brain slice are basic neural models for in vitro patch-clamp recording. With the development of patch clamp technique, the in vivo patch-clamp recording is also applied in the neuroscience study [261–265]. In contrast to in vitro recording, the advanced in vivo patch-clamp recording paves a new path for neuroscience of living organism. In vivo patch-clamp recording is commonly performed under a microscope to find and record the labeled neurons in the brain. To simplify the pre-labeling of neuron, Kitamura et al. developed a novel shadow patching method to visualize the neuron as a negative image by prefusing fluorescent dye in the extracellular environment (Fig. 16a, left) [261], and the recorded action potentials present the similar quality with the pre-labeled one [266]. Moreover, the plasmid DNA and fluorescent dye was also successful to be delivered into the neuron by electroporation of the same platform, which facilitates the signal recording, biomarker labeling, and genetic manipulating of single neuron in intact brain. Although the in vivo patch-clamp is a powerful tool to study the electrophysiology of single neurons, the manipulating skills are strictly required. To achieve the user-friendly operations, an automatic in vivo patch clamping system (Fig. 16a, right) was established to accurately position the cell by analyzing the cell-induced electrode impedance changes in algorithm with good performance of yield, throughput and signal quality [262]. Although the intracellular recording of single neuron is fundamental to understand the neurophysiology, the single-electrode system with complicated components is difficult to scale to a high-throughput system, and the invasive manners hamper the development of long-term recording.

For chronic neurophysiology study, electroencephalograph (EEG) recording featured with noninvasive and high-throughput properties is widely applied to record local electrical activities of brain, which is collected from the multiple electrodes on the scalp [42–44, 270, 271]. However, the spatial resolution is limited due to the electrode size, and the quality of EEG recording is also affected by the dura and skull [272–274]. To improve the spatiotemporal resolution of recording, the extracellular microelectrode arrays (MEAs) are designed to record the neuronal action potentials from local neuron network. In contrast to EEG recording, the semi-implantable MEA extracellular recording can not only distinguish signals (e.g., spike) from individual single neurons in local neural circuit, but stimulate single neurons in the deep brain as well. Significantly, the high spatial resolution MEA can map and explore the working mode of neuron network [275–277].

Microwire MEAs are the first semi-implantable brain electrodes to study the brain for decades (Fig. 16b) [278–283]. Microwires are manually made of insulated material coated stainless steel, tungsten, platinum, gold, iridium or nichrome wires with exposed conductive recording tips. Conventionally, the microwires are categorized into tetrodes and stereotrodes with throughput from several to over hundred electrodes, which are collected in a guide tube or aligned along the socket, respectively. The conductive tip end will be penetrated into the brain, and the other ends of microwire is soldered to a connector for signal recording [267, 284]. Much effort was made to long-term single neuron recording from the mammalian by microwires. To further improve recording sites in the brain, multiple boards can be stacked up to form a high-throughput microwire MEAs. Nicolelis et al. implants 96–704 microwires in monkeys, recording 421 single neurons for one month and 58 neurons for 18 months after implantation [284]. Due to less damage to the neurons, the microwire MEAs are qualified for the chronic, large-scale electrophysiological recording of mammalian [285–287]. However, the soft microwires are difficult to control during implanting into the curved brain surface.

With the development of micro-electromechanical system (MEMS) technologies, the complicated silicon-based MEAs emerge by efficient and standard microfabrication techniques. Utah electrode array (UEA) is similar with the stacked microwire MEAs, and its conventional configuration contains 10-by-10 needle shank with the exposed conductive silicon tips, which is originally developed at the University of Utah (Fig. 16c) [288, 289]. UEA is fabricated on a 3-mm silicon substrate, which make it a robust semi-implantable device in mammalian research [268, 290, 291]. Moreover, the successful human trials are usually based on this type of MEAs using brain–machine interface [292, 293]. Compared with the stacked microwires, UEA has a higher spatial resolution at the same brain depth, and the pitch between neighbor shanks is standard. To further improve spatial resolution in-depth, Utah slanted electrode array (USEA) is developed to detect the neuron signals at different depth [294, 295]. However, the spatial resolution in-depth is still limited due to intrinsic structure of UEA or USEA. To realize the recording from various depth of brain, Michigan electrodes (Fig. 16d) with single or multiple shanks are effective complement and development for functions of microwires and Utah electrodes, and Michigan fine neural probe was defined and released by deep reactive ion etching (DRIE) on silicon-on-insulator (SOI) wafers [296, 297]. In contrast to tip recording sites of other two MEAs, Michigan electrodes have multiple recording sites along each shank for simultaneously exploring the neuronal activities at different brain layers, which greatly improve the depth spatial resolution [269, 298, 299]. Besides, Michigan electrode can be easily assembled as UEA by stacking the multiple-shank electrodes for three-dimensional high spatial resolution recording, while the recording brain depth (e.g., cortical or intracortical) is adjusted by the shank length [300].

**Fig. 17:**
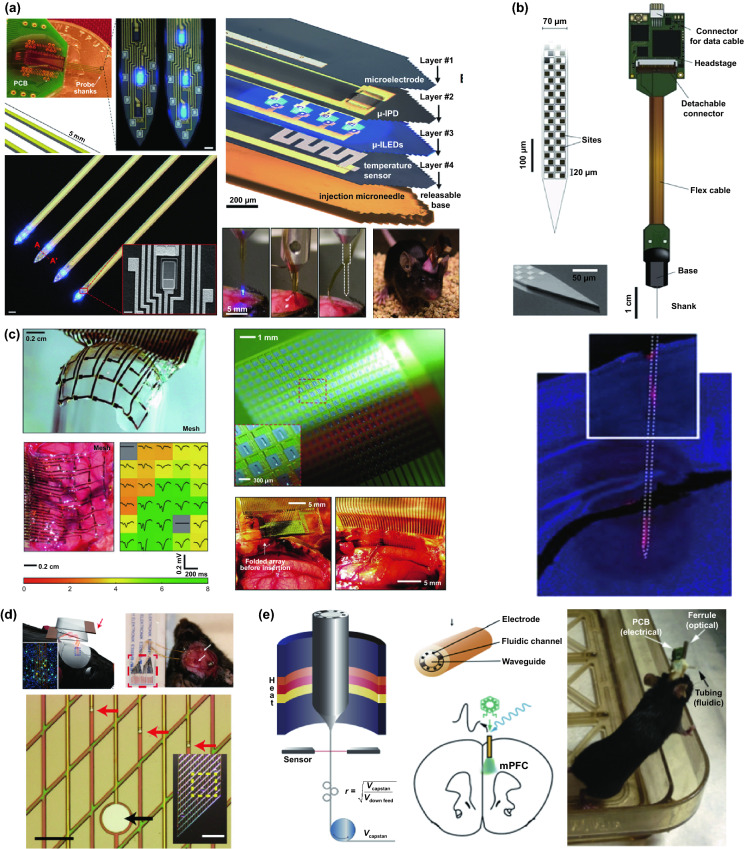
**a** Optical component integrated multifunctional Michigan electrodes for optogenetic applications [301, 302]. **b** Microchannel integrated Michigan electrodes for drug delivery (Top) and CMOS-based high-density recording of neural activities (Bottom) [303]. **c** Flexible electrode array for ECoG and intracortical signal recording [304, 305]. **d** Injectable mesh electrodes for long-term brain mapping and neural recording [306]. **e** Multifunctional FEAs for simultaneous optical stimulating, electrical recording, and drug delivering of neural circuit in vivo [307, 308]. Reproduced with permission from Refs. [301–308], copyright (2015) Cell Press Publishing Group, (2013) Science Publishing Group, (2017) Nature Publishing Group, (2010) Nature Publishing Group, (2012) Nature Publishing Group, (2016) Nature Publishing Group, (2015) Nature Publishing Group and (2017) Nature Publishing Group

Based on the high spatial resolution and controllable fabrication of Michigan-style electrodes, other functional components can be integrated into the same device. In neuroscience, neuronal regulation is another important approach to reveal neurons’ functionality and interaction in complex networks [309, 310]. Electrical stimulation is preferred to activate neurons due to the sensing electrodes can be multiplexed as a stimulating one, reducing the fabrication and integration complication of devices. However, the electrical stimulation has the disadvantages of lower spatial resolution and non-specificity [311]. To solve the limitation of electrical stimulation, optogenetics provide an advanced neuronal regulation strategy using specific wavelength optical stimulus at the neurons which are introduced the photosensitive proteins, and this novel optical simulation strategy can improve the neuron network analysis by the high spatiotemporal stimulation. According to this principle, the high-density microscale optical and optoelectronic components trend to be integrated on the Michigan-style probe by advanced MEMS technologies. To control distinct cells and field oscillations in animals, Wu et al. fabricated the neuron-size microscale light-emitting diodes (μLEDs) and recording electrodes on the same neural probe shank (Fig. 17a, left), demonstrating the versatile and precise optical simulation by this optogenetic tools [301]. To meet the freely moving animal applications, the wireless optogenetics was developed by Kim et al. [302], and the neural probe involved electrophysiological measurement (Pt microelectrode), optical measurement (Si microscale inorganic photodetector), optical stimulation (GaN inorganic light-emitting diodes), and temperature sensing (Pt serpentine resistor) on a microneedle substrate for injection into the brain (Fig. 17a, right). In addition to the MEMS μLED integrated device, the optical waveguide integrated probe is another optogenetic tool to deliver the external coupled optical source to the neural probe [312–314]. The waveguide can be fabricated by polymers such as SU-8 or dielectrics, and the path and stimulation sites of waveguide microscale pattern can be freely defined by photolithography [312, 315–317]. In addition to the electrical and optical stimulation, the chemical stimulation is also an alternative mean to regulate neurons in brain, so the microfluidic channels (Fig. 17b, top) were integrated on the neural probe shank to achieve simultaneous electrical recording and drug delivery in deep brain [47, 318–322]. Although the Michigan-style electrodes have high-density recording sites, the interconnection lines of passive probe arrays significantly limit the number of electrodes on each shank. Owing to complementary metal–oxide–semiconductor (CMOS) devices are addressable by multiplexing circuits via a small number of interconnection line. Therefore, the number and spatial resolution of recording sites (Fig. 17b, bottom) can be sharply improved by these active devices [274, 303, 323–325].

The silicon or metal-based electrodes are popular and utility tools for the neuronal signal recording; however, they also suffer from the mechanical mismatch with the soft brain tissue, leading to the chronic immune responses. To improve the neural probe and brain tissue interface, the flexible electrode arrays (FEA) are designed by the materials, (e.g., polyimide (PI), parylene, or SU-8) or configurations (e.g., ultrathin mesh) with the low Young's modulus and bending stiffness, achieving a high-quality and biocompatible coupling with brain tissue. For example, Kim et al. fabricated FEA on ultrathin polyimide substrate supported by bioresorbable substrates of silk (Fig. 17c, left), demonstrating 2.5-μm mesh PI-based FEA enabled conformal contact with soft curvilinear brain tissue. All the electrodes record the high-quality electrocorticogram (ECoG) without implantation immune response for 3–4 weeks [304]. To overcome the constraint of passive electrode interconnection line, Viventi et al. developed a ultrathin and flexible silicon nanomembrane transistors with thousands of recording sites [305], and this FEA device can be either placed on the brain or folded and inserted into interhemispheric fissure (Fig. 17c, right). FEA paves a utility tool for long-term continuous monitoring and coupling tightly with uneven brain surface, remaining precise delivery difficult in deep brain. To overcome this challenge, syringe injectable electronics was propose by Liu et al. [326]. The mesh FEA was delivered via 100 μm needle to achieve the tight integration in distinct regions of the brain with high device yield (> 90%) and low chronic immune responses in five weeks (Fig. 17d, left). For stable long-term chronic brain mapping, the mesh FEA was further designed with a lower bending stiffness close to one of neural tissue, and the injectable in vivo recording lasted for at least 8 months (Fig. 17d, right) [306]. To enrich the functions of FEA, the optical waveguide, recording electrodes, and microfluidic channels were integrated by fiber drawing process, and this device simplified the multiple optogenetic steps to single step (Fig. 17e) [308]. Via same drawing process, high-throughput and multifunctional fiber probes simultaneously carried out the optical stimulation, drug delivery and neural recording and drug delivery in freely moving mice with high-resolution study.

Bioresorbable materials are introduced in the semi-implantable device to enable the reliable implantation and good biocompatibility. Bioresorbable material-based electrodes provide another biocompatible strategy in neuroscience study. The key of these electrodes is the application of bioresorbable materials used as the thin substrate film or coating components, which can seamlessly biodegrade in the tissues over time [327–329]. Bioresorbable polymer coating (e.g., polyvinyl alcohol and poly(lactic-co-glycolic acid)) has been demonstrated to reduce glial scarring during the insertion of semi-implantable devices [327]. Bioresorbable silk coatings are an effective temporary stiffening agent for the soft polymer-based probes which are difficult to be implanted into the brain, and silk will gradually in the brain tissue [330].

In addition to organic materials, inorganic materials such as monocrystalline Si nanomembranes are demonstrated to fabricate a bioresorbable array [331]. These Si nanomembranes are hydrolyzed into biocompatible products (e.g., silicic acid) when immersed in the biofluidic environment. The 300-nm-thick phosphorous-doped Si nanomembrane dissolves with the rate of 11 nm/day in 37 °C and pH 7.4 artificial cerebrospinal fluid, while the rates of 100-nm-thick passivated SiO_2_ layer and 30-µm-thick PLGA are both 8.2 nm/day, respectively. Significantly, in vivo biocompatibility bioassays verified these bioresorbable devices activate small-scale microglials. Those study proves the bioresorbable semi-implantable devices serve as the brain exploration tools in comfortable and biocompatible manner based on the highly precise microfabrication technologies. To lower the stiffness of electrodes, the low-modulus conductive polymers are adopted in the neuro-device fabrication to provide a good mechanical match for brain tissue [332]. Poly(3,4-ethylenedioxythiophene) (PEDOT) is a common conductive polymer, which can replace the gold, platinum, iridium, and tungsten materials as recording and stimulation electrodes. The PEDOT/poly (styrene sulfonate) (PSS) recording electrodes are widely reported in many studies, while the PEDOT/paratoluene sulfonate (pTS) is preferred material as the stimulation electrode. PEDOT/pTS-coated platinum electrodes exhibit a lower impedance and larger charge injection capacity than those of bare ones [333]. Due to the higher surface area, these conductive polymer-based electrodes present a higher charge transfer capacity for the in vivo models [334]. To future clinical applications, these conductive polymer-based semi-implantable devices require more rigorous validation in nonhuman primate models to ensure the chronic stability and biocompatibility [335].

### Applications

**Fig. 18:**
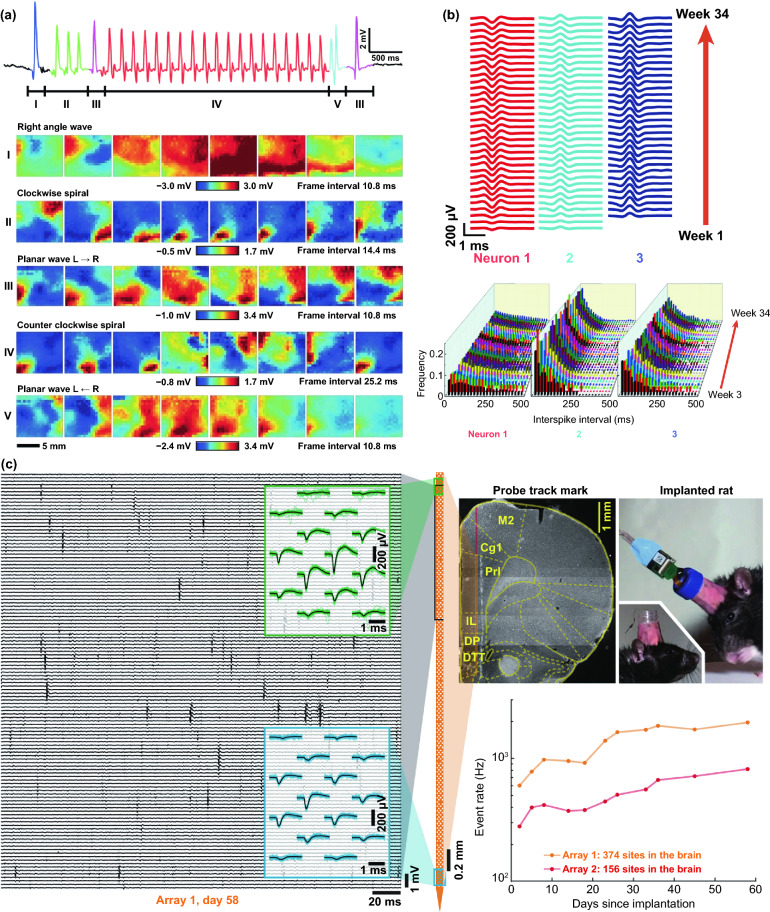
High-throughput and long-term neuroscience study by semi-implantable devices. **a** High-throughput µECoG signal recording by 360 transistor-based arrays during a short seizure. Transistor array can visually display the high spatiotemporal resolution movie frames in various patterns [305]. **b** Long-term chronic single-neuron recording by neuron-like semi-implantable device for 34 weeks [306]. **c** 960 CMOS semi-implantable device for high-throughput and long-term recording [303]. Reproduced with permission from Refs. [303, 305, 306], copyright (2017) Nature Publishing Group (2012) Nature Publishing Group and (2016) Nature Publishing Group

For the fundamental neuroscience research, electrical recording is a powerful technology to analyze the activities of neural networks, which are related to physiological and biological mechanisms. MEAs are commonly employed semi-implantable devices for high-throughput and long-term electrophysiological recording. The traditional MEAs consist of multichannel individual passive sensors; however, their interconnectors occupy large space, which significantly limited the high spatial resolution for the in vivo neuroscience studies. Viventi et al. employed a silicon nanomembrane-based transistor array for the ECoG recording to overcome this limitation [305]. Based on architecture of the flexible transistor array, the thousands of integrated sensors can be operated by fewer wires in a multiplex way. The in vivo activities of brain cortex, such as sleep, visual stimulus, and seizure, were recorded and mapped by 360 electrodes. From the feline seizure model, it can be found the typical feature of seizures is spiral waves which propagate in the cortex, and the high spatial resolution devices can visually display in the form of high spatiotemporal resolution pseudo-color movies (Fig. 18a).

For brain electrodes, fully implantable electronics facilitate the maximum free movement of the subject without the influence of external structures; while semi-implantable electronics focus more on the fidelity of signal transmission. Since fully implantable electronics need to meet the requirements of wireless transmission, and the current development of fully implantable electronics, the wireless transmission requirements of fully implantable electronics are far from the expected goal, especially the EEG signal and its complex and cranial will seriously affect the transmission of the signal. In addition to the high-throughput property of devices, long-term chronic electrical recording is also required in neural electrophysiological study. For the stable in vivo neuroscience investigation, the semi-implantable device can achieve the single cell spatiotemporal resolution; however, the motion damage and chronic inflammation will affect the function and performance of implanted probes during long-term monitoring. Fu et al. fabricated a flexible mesh electronic device, which was biocompatible with neuron-like stiffness to support the high-quality neuron signal recordings from mouse brains for 34 weeks (Fig. 18b) [306]. The neuron-like device can record the robust single-neuron signals from freely behaving mice, which paves a promising way to investigate the cognition and neurodegenerative in vivo models. Therefore, under the premise of requiring signal fidelity, the current semi-implantable electronics are more widely used in the field of brain electrodes.

To integrated high-throughput and long-term properties of neural activity recording, the fully integrated silicon probe was developed for the high-density recording. The Michigan-style shank integrated 960 complementary metal–oxide–semiconductor (CMOS) recording sites (Fig. 18c), which can perform the hundreds of neuron for each semi-implantable device [303]. Employing only two shanks, at least 700 individual neurons were recorded from five brain regions in a freely moving mouse. Moreover, more than 100 neurons were stably chronic recordings over 150 days by a semi-implantable COMS device in the mouse brain. Significantly, the functionalized small devices allowed the simultaneous and large-scale recording from different brain regions of awake animals. Combing the high-throughput and long-term performance, this advanced brain electrodes open up future study of large-scale ECoG recording in both local field potentials and spikes.

#### Biochemical Sensing

Due to the existence of skull structure, it is not suitable for the detection of brain biochemical indexes with common transdermal detection. Discoveries in optical sensing [45, 336, 337] and electrochemical sensing [338–341] are of great importance to the development of neuroscience.

**Fig. 19:**
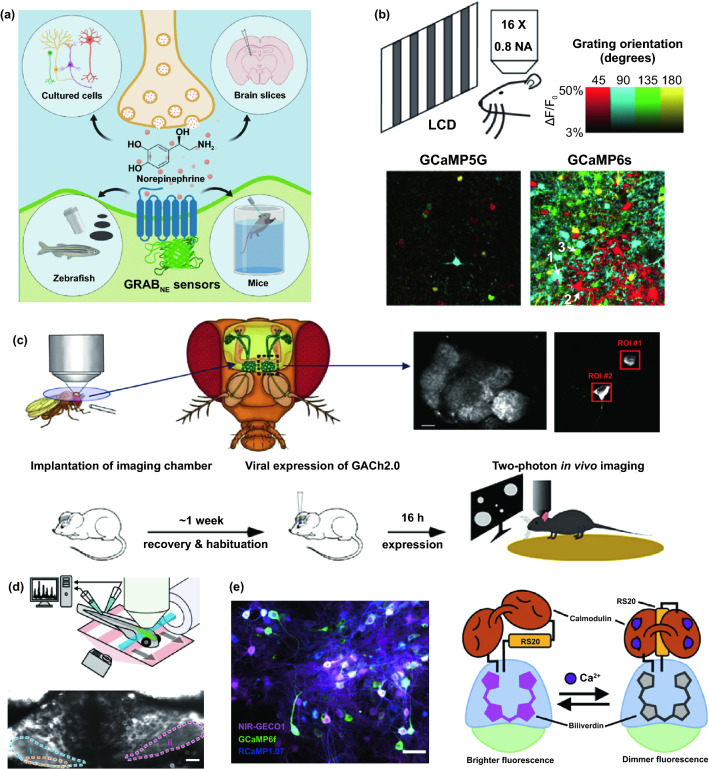
Semi-implantable optical sensor for brain in vivo. **a** Genetically encoded GPCR-activation-based on norepinephrine sensors for rapid in vivo specific detection for norepinephrine [342]. **b** In layer 2/3 pyramidal neurons of the mouse visual cortex, Ultra-sensitive protein calcium sensors (GCaMP6) detected of single-neuronal somatic action potentials and targeted regulation of synaptic transient calcium in single dendritic spines [343]. **c** G-protein-coupled receptor activation-based ACh (GACh) sensors responded to exogenous and/or endogenous ACh selectively with remarkable fluorescence signals that were recorded by epifluorescence, confocal and/or two-photon microscopy from multiple animal species [344]. **d** Genetically encoded GABA fluorescent sensor for in vivo light-sheet image of zebrafish cerebellum expressing [346]. **e** Spectral multiplexing of genetically encoded near-infrared fluorescent calcium ion indicator NIR-GECO1 with optogenetic indicators and actuators [337]. Reproduced with permission from Refs. [337, 342–344, 346], copyright (2019) Nature Publishing Group (2019) Cell Press Publishing Group, (2013) Nature Publishing Group (2018) Nature Publishing Group and (2019) Nature Publishing Group

Most of the initial optical sensing was based on single-electrode sensing of fluorescent proteins. This single-electrode optical biochemical sensor provides a solution for in vivo research in neuroscience and lays the foundation for the development of modern precision electrodes. For example, GRABNE single-electrode sensor detects optogenetically and behaviorally triggered NE release in live mice, live zebrafish, and free-ranging mice (Fig. 19a) [342]. As shown in Fig. 19b, the ultra-sensitive protein calcium sensor GCaMP6 reliably detects individual action potentials in neuronal cytosol and directionally regulated synaptic calcium transients in individual dendritic spines [343]. Thus, the optical sensor provides a new avenue to the organization and dynamics of neural circuits at multiple temporal and spatial scales. With the development of single-electrode imaging, two-photon imaging [344] and high-precision and multifunctional [337, 345, 346] imaging are maturing. The easy detection characteristics of these versatile, high-precision optical sensors make it possible to monitor neural activity in vivo in real time. Acetylcholine, which is involved in a variety of neural activities but has always been difficult to detect, was successfully detected in vivo by a semi-implanted fluorescent, two-photon imaging device (Fig. 19c). As shown in Fig. 19d, the behavior of acute GABA release can be monitored, and the amount of instantaneous change in dopamine can be measured. In terms of application prospects, wavelength division multiplexing sensing of optical sensors will be applied to more scenarios. A calcium-sensitive near-infrared probe (NIR GECO1) combined with other optogenetic indicators and actuators has been reported to open up new prospects for multicolor Ca^2+^ imaging (Fig. 19e). In the future, it will be a trend to use wireless, passive, multiplexed implantable optical sensors to monitor and modulate neural activities in freely moving animals. The important progress in brain sensing was also extended to the development of electrochemical (EC) sensors.

**Fig. 20:**
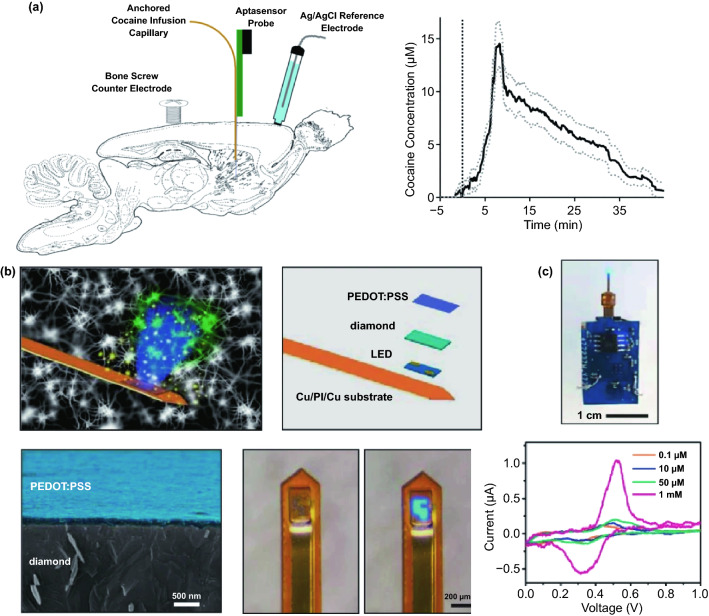
Semi-implantable electrochemical sensor for brain in vivo. **a** (left) Representative sagittal plots of in vivo experimental designs for cocaine detection experiments. (right) In vivo detection of systemic I.V. cocaine injection [348]. **b** Schematic diagram of an implantable microprobe. **c** Photographs of implantable probes and CV curves in HCl solutions with different dopamine levels [347]. Reproduced with permission from Refs. [347, 348], copyright (2020) Nature Publishing Group and (2017) The Royal Society of Chemistry Publishing Group

Similar to common electrochemical transdermal sensing, electrochemical sensing in brain relies on working electrode and electroanalytical techniques. Micro-/nano-electrode modified by aptamer has become a main way to detect biochemical substances in brain. For example, it was reported an electrochemical aptamer-based in vivo cocaine sensor capable of measuring cocaine directly from discrete brain locations. Besides, aptamer functionalized neural recording electrodes successfully probe electrochemical aptamer-based sensor and spontaneous neural activity in the brain (Fig. 20a). Another electrochemical aptamer-based sensor supporting continuous, real-time, multi-hours measurements detected four drugs in the bloodstream of even awake, ambulatory rat (Fig. 20b, c). This electrochemical aptamer-based sensor achieved high temporal resolution and precise molecular measurements at clinically relevant detection limits, providing an important approach to the study of physiology and pharmacokinetics. A recent paper describes a transparent, ultra-flexible, and active multielectrode array, for simultaneous optogenetics and electrochemical sensing. With the increasing of multifunctional semi-implantable sensors in brain biochemical detection, wireless multifunctional devices are booming. A wireless miniaturized microelectrode system for real-time optogenetic stimulation and dopamine detection in the deep brain of freely behaving mice was developed [347]. Such semi-implantable biochemical sensors provide important potential for neuroscience studies when combined with fluorescent and aptamer for mapping of biochemically active species.

#### Drug Delivery

**Fig. 21:**
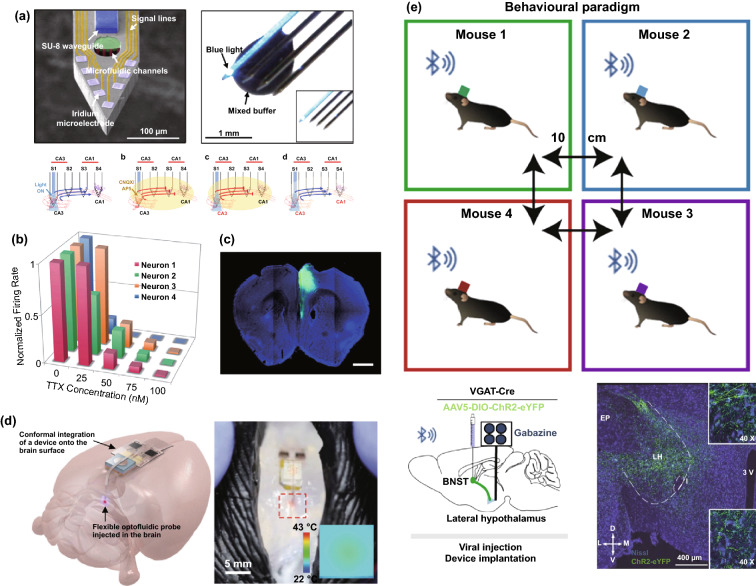
Semi-implantable drug delivery devices for brain in vivo. **a** A multifunctional multi-shank with single channel microfluidic for drug delivery neural probe to study and modulate remote neural circuits in vivo [349]. **b** In vivo direct injection of pilocarpine recording that four sorted neurons at 5 different concentrations of pilocarpine by microfluidic channels [47]. **c** The expression of opsin gene in wild-type (WT) mice was detected after the viral vector carrying opsin gene was injected into WT mice by dual channel microfluidic probe [350]. **d** Wireless delivery of red dye to the phantom brain in the rat model by single channel optofluidic system [351]. **e** Wireless, selective control of drug delivery in a group of concurrently behaving mice [352]. Reproduced with permission from Refs. [349–352], copyright (2019) Nature Publishing Group, (2015) The Royal Society of Chemistry Publishing Group, (2017) Nature Publishing Group, (2018) Wiley-VCH Publishing Group and (2019) Nature Publishing Group

Advances in the field of nano- and microfluidics have created opportunities to develop drug delivery platforms in a semi-implantable form. The advanced microfluidic systems allow precise temporal control of variety drugs, long-term independent infusion with minimal tissue damage and are designed to allow free movement of research animals. In recent years, drug delivery systems are often used in combination with multifunctional platforms such as photo-modulation and signal recording, and drug delivery channels have evolved and improved from single to multiple channels depending on the application requirements. As shown in Fig. 21a, different stimuli are applied at the cellular level to study and modulate neural circuits in vivo, which only a single microfluidic channel is used for drug delivery. In the example shown in Fig. 21b, neural electrodes with multiple functions are also capable of delivering multiple drugs. These in vivo results demonstrate that these electrodes with multifunctional delivery capabilities will be an effective tool for investigating the fundamental mechanisms behind brain disorders that modulate neural circuits by delivering neurotransmitters through a single implant. Multifunctional electrodes like the aforementioned one, which contain multiple drug delivery channels, can deliver not only common neurological drugs but also vector viruses carrying special genes (Fig. 21c). These flexible electrodes, which have multiple functions such as drug delivery, optical pathways, and signal recording, have achieved high fidelity (similar to the transmembrane potential and contains high-resolution details to explore ion channel properties) multimodal interrogation of brain circuits while minimizing tissue damage. But for accurate drug testing to be done in a free-moving, awake animal, smarter and easier drug delivery systems will be needed. Examples of wirelessly implantable drug-deliverable electrodes are shown in Fig. 21d-e. Figure 21d shows this wireless electrode can analyze dose response relationships for a single drug, or test the effects of several various drugs in a rodent brain. The main difference is the drug-fillable and flow-adjustable feature. The technique has the capability to target specific neuronal populations in freely moving animals. For the more demanding requirements of wireless, prolonged, repeated and precise drug delivery, the multichannel, wireless, controlled drug delivery shown in Fig. 21e provides a reliable solution. This scheme demonstrates the optofluidic device is an ability to selectively control specific mice via a Bluetooth application for smartphones, revealing the basis of neuropsychiatric disorders by observing changes in behavior and intracerebral features of the mice.

#### Stimulation

Modulating the activities of neural networks plays an essential role in neuroscience. Electrical and optical stimulation are common approaches to regulate the activities of neurons. Brain stimulation electrodes that are not permanently implanted inside the brain can be classified as semi-implantable devices because of their non-permanent use and because they can be controlled by an external device. To address the disadvantages of low spatial resolution and non-specificity, brain stimulation electrodes can be combined with optogenetics and advanced MEMS technology to activate neurons. It is of interest that deep brain stimulation (DBS) is used to treat neurological disorders such as Alzheimer's disease (AD), Parkinson's disease, or depression. Currently, the main focus is on the versatility of semi-implantable devices to achieve closed-loop recording and treatment with DBS. Due to the excitability and conductivity of neurons, the electrical stimulation is generally applied to regulate them. The electrical stimulation electrodes are usually integrated in the electrical recording devices to check electrophysiological properties of neurons. These multifunctional semi-implantable devices can provide a simultaneous stimulation and recording tools for long-term monitoring and evaluation of single-neuron activities [306]. The emergence of optogenetics modified the gene of neurons into photosensitive ones which facilitates the optoelectronic regulation and greatly propels the neural regulation [353–355]. In contrast to the conventional electrical stimulation, optical stimulation can dramatically reduce the interference while signal recording is simultaneously performed. In the early research, the LEDs were physically attached on the brain recording probe with low resolution [356]. Recently, more efforts are focused on the integration of microLEDs on the neural probe with advanced microfabrication for the high-resolution optogenetic modulating and recording [301]. In recent study, the gamma frequency ontogenetic stimulation has been proved a hopeful DBS for AD treatment. It is demonstrated that 40 Hz optogenetic stimulation reduce the amyloid-β levels, and the microglia cells are activated to help clear amyloid-β.

To perform the optical stimulation, some studies have employed the thin optical fibers and waveguides with recording probes. For example, this optical fibers-microwire hybrid probes can achieve the simultaneous recording of single-neuron responses to the optical modulation. To further improve the resolution of optical modulation, the tip sizes of optical fibers were reduced to 5–20 µm to form an integrated optoelectrical interface [46, 357]. Another group has developed small and good flexible multifunctional probe by optical waveguides and recording electrodes, and this polymer-based multifunctional probes can be applied for optoelectronic operation on neural activity in the mouse brain [307, 308]. In one study, transparent and conductive zinc oxide pillars-based brain probe simultaneously performed the signal recording and optical regulation of optogenetic modified neurons in cortical regions. Based on this semi-implantable device technology, dynamics of light-perturbed brain circuit of transgenic mice and effects on behavior can be in-depth investigated [358].

In addition to the electrical and optogenetic stimulation, neural activities can also be modulated by chemical stimulation. For this purpose, the microfluidic channels were integrated onto the brain probes by deep reactive ion etching, and these channels were connected the exterior delivery of various neuroactive biochemical. In a study, the multiplex delivery microchannels were designed for simultaneous infusions of a stimulating chemical, a saline buffer solution, and label chemical into a local mouse brain for modulating neural activities and staining neurons at the target area. As a result, the neural activities were enhanced by the infusion of pilocarpine, while significantly inhibited by infusion of tetrodotoxin [47]. Moreover, the delivery function integrated neural probes were demonstrated to establish the in vivo disease model. By injection of baclofen, the seizure models can be built and specific neural signal patterns can be recorded simultaneously [47].

### Biosafety

The biosafety indicators of semi-implantable devices are particularly concerned in both clinical practice and biomedical studies, mainly including inflammation, irritation, and fibrosis evaluation. Inflammation is a common defense mechanism in the body to eliminate harmful stimuli and activate the healing, when the immune system finds the pathogens, damaged cells, or irritants. However, inflammation sometimes can last longer than necessary, causing unexpected harm to the body. The effects of acute inflammation can be summarized as pain, redness, immobility, swelling, or heat signs, which only apply to inflammations of the skin. Irritation is defined as an inflammation or painful response status, which results in allergy or cell damage. For example, once the stimuli or agents induces the skin irritation, the skin may turn red. Fibrosis is another biosafety indicator, which is the formation of excess fibrous tissues or scar tissue, due to the injury or long-term inflammation. The formation of fibrosis has many potential causes such as injuries, burns, radiation, diseases, or treatment of diseases. The negative effects of fibrosis make the tissues harden or swell, which significantly affects the normal function of tissues.

Subcutaneously implanted electrodes or probes (e.g., CGM sensors) generally damage cells or stimulate skin, which will induce the inflammation. The early implantation has been proved to cause of sensor noise rather than the fibrosis [359]. The foreign-body response and wound injury will not lead to significant inflammation with insertion and immediate removal of the guide needles [177]. However, the implanted position is usually selected at fat region of abdomen, where the friction or touch will frequently occur during the sleeping or movements, so the long-term implantation of electrodes will result in subcutaneous inflammation, which will disturb the accurately blood glucose measurements. Besides, the tubing of insulin pump is easily blocked due to the similar reason, resulting in the dangerous hyperglycemia. For the brain electrode implantation, the foreign body response also leads to the wrapping of glia cells [359]. Generally, glia cells have been recognized as an encapsulating barrier and directly affect the communication between the implanted electrode and targeted neurons. Consequently, the glia will significantly influence the stimulation efficacy and the long-term performance of implanted devices. Due to the above reasons, the CGM sensors can only be implanted for 2 weeks to clean the probes, while the insulin pumps can last for 1 week to avoid the blockage. The brain electrode can continuously maintain the high-quality electrophysiological signals for several months, while the most of microneedles sensing and regulating system only present short-term applications.

### Limitation and Future Trends for In Vivo Applications

Semi-implantable devices (e.g., CGM-based insulin pump systems, microneedles, brain electrodes) possess advantages of high sensitivity and rapid response for recording and regulating compared with the wearable devices. The electrically controllable property is convenient and reliable for the data transmission between the device and computer. In contrast to the fully implantable devices for in vivo application, the semi-implantable devices are safe without residues and convenient for power supply. For the in vitro cell application, the semi-implantable devices (e.g., nanoelectrode array, nanoFET, nanowire, and nanostraw) are efficient to penetrate the cell membrane in spontaneous or artificially assisted way. Due to the superiority of semi-implantable devices, the high-quality intracellular biophysical and biochemical signals can be recorded accurately, while the intracellular drug delivery can be precisely operated.

On the other hand, semi-implantable devices have obvious defects, such as limitation of long-term implanted, space for the in vitro intracellular environment, and imaging effect. Compared with the wearable devices, the long-term fixation on the human body causes inconvenient and uncomfortable feeling. Most of semi-implantable devices cannot be chronically implanted, which is easy to induce the inaccurate measurement or cause inflammation. Moreover, requirement of multiparameter simultaneous detection is difficult to be satisfied due to high standard for in vivo clinical practice and limit space for the in vitro intracellular environment. Furthermore, the detection sensitivity and selectivity of devices need to be improved. Spatial resolution and imaging effect is still at a low quality.

## Summary and Perspective

Semi-implantable bioelectronic devices are desired to extensively promote the personalized and precise healthcare. They present the potential to serve as powerful tool for the biomedical clinical practice with the characteristics of high sensitivity, high selectivity, good biocompatibility, etc. With advances of micro/nanofabrication technologies, chemical/material preparations, and intelligent control algorithms/systems, the high-performance semi-implantable bioelectronic devices can sense and regulate the trace fluctuations (e.g., biophysical signals and biochemical markers) of physiological status. Microfluidics is another prospective technology for drug delivery and signal monitoring in combination with nanoelectrodes. The combination of electrical stimulation and nanoelectronics can modulate cellular activity and expression for precise stimulation and simultaneous monitoring. For personalized and precise healthcare, the universal semi-implantable bioelectronic platforms are compatible for various individuals, while the closed-loop regulation strategies can also be self-adaptive for individual requirements by artificial intelligence algorithms. For the in vitro cell applications, semi-implantable bioelectronics shows the superiorities of intracellular accurate signal recording and precise manipulations in a minimally invasive way, which collected high-quality intracellular bioinformation and achieve the efficient intracellular delivery.

In the recent decades, semi-implantable bioelectronic devices have unprecedented development in a variety of biomedical fields. To propel the wide practical applications of these semi-implantable bioelectronic devices, a large amount of current challenges and bottlenecks should be positively addressed for the future wide research and development. Here, we prospect the six future research trends for semi-implantable bioelectronic: (i) multifunctionality, (ii) microminiaturization, (iii) biocompatibility, (iv) intelligentization, (v) reliability, and (vi) commercial practicality. Multifunctionality is future trend in developing the semi-implantable bioelectronics. Multifunctional devices will not only perform the multiplex biochemical and biophysical signal recording, but conduct the electrical, optical, chemical regulating as well in vivo and in vitro. To explore the diversity and complexity of biological living from cell and tissue to living body, from extracellular environment and intracellular environment, the multifunctionalized semi-implantable bioelectronic devices are powerful tools to carry out the multiplexed signal collections for the multi-modality investigation.

Even if the current devices are miniaturized into a portable size, microminiaturization of the next-generation semi-implantable bioelectronic devices further helps the users ignore the existence of them. The microminiaturization with invisible properties should be developed with improved humanized design by integrating microminiaturized devices into daily necessities without uncomfortable implanting/carrying feelings. Meanwhile, the microminiaturization of semi-implantable devices for cells will greatly improve the chronic intracellular investigation with minimal damage.

For the long-term applications in vivo or in vitro, biocompatibility is one of key properties in the semi-implantable devices. For the in vivo studies, the materials of should be strictly screened to relieve the foreign body response, and the suppressive negative effect of semi-implantable devices will prolong the working life and reduce the recalibration frequency. For the in vitro studies, the biocompatible semi-implantable devices will enter into the cell and monitor the intracellular environment by a spontaneous and gentle endocytosis rather than artificial operations, which effectively maintain the cell viability and integrity of plasma membrane.

Though these semi-implantable devices can automatically perform regulation on biological subjects based on the collecting information, development into highly intelligent and close-loop system is still further required for personalized and precise healthcare. By integrating the artificial intelligence algorithms, the semi-implantable bioelectronic platforms are compatible and universal for various individuals. The closed-loop regulation strategies can also be supportive for individual requirements based on the precise deep learning or self-adaptive algorithms embedded into the control system of device.

Reliability of semi-implantable bioelectronic device is also essential aspect for the practical applications. The accuracy and precision of the measurement play a crucial role in the practical use, particularly in rigorous clinical trials. The fundamental performance, such as selectivity, sensitivity, long-term stability, and noise suppression, of semi-implantable bioelectronic devices should be further tested to assure an accurate measurement and regulation to exclude the interferences (e.g., non-targeted biochemical or biophysical signals, background noise). Reliability of devices should be also based on the further development toward biocompatibility and high-performance, while taking the maintenance of multifunctionality and device miniaturization into account.

With the advanced properties of semi-implantable bioelectronic device, the commercial practicality is leap development from laboratory to industry, or even from bench to the clinic. The inaccuracy or uncomfortable experiences of commercial semi-implantable bioelectronic devices will induce the distrust on its practical applications, which will rapidly cause the failure of commercial products. The translation from the prototype of semi-implantable electronic device to the commercial practical device suffers from the extremely high requirements and enormous challenges. However, for the future advances of biomedical practical application, these challenges of semi-implantable bioelectronics are meaningful to be achieved. The versatile semi-implantable bioelectronics will eventually provide the profound benefit of mankind from daily life to personalized healthcare.
